# Spatially resolved single-cell transcriptome analysis of murine *Salmonella* infection reveals the role of distal colonocytes in the inflammatory response

**DOI:** 10.1080/19490976.2025.2579909

**Published:** 2025-11-24

**Authors:** Dan Xu, Ruifen Zhang, Shanshan Li, Can Guo, Chenglin Guan, Xiang Li, Mengyao Guo, Xin Xu, Yaxin Liu, Chenyi Mao, Peisen Sun, Xiaomin Dang, Diya Sun, Chengyao Wang, Stephen J. Bush, Kai Ye

**Affiliations:** a Key Laboratory of Biomedical Information Engineering (MOE), School of Life Science and Technology, Xi’an Jiaotong University, Xi’an, China; b School of Automation Science and Engineering, Faculty of Electronic and Information Engineering, Xi’an Jiaotong University, Xi’an, China; c The First Affiliated Hospital, Xi’an Jiaotong University School of Medicine, Xi’an, China

**Keywords:** *Salmonella*, intestinal compartmentalization; DCCs (distal colonocytes), host response, enteric inflammation

## Abstract

The intestine is a highly compartmentalized organ, with distinct segments exhibiting both varying susceptibilities and responses to enteric pathogens, although the cellular and molecular bases of these responses remain elusive. Here, we used *Salmonella* Typhimurium (S. Tm), a prominent enteric pathogen that causes human colitis, to establish a murine model of *Salmonella* enterocolitis. By integrating bulk RNA-seq, single-cell RNA-seq, and spatial RNA-seq data, we present a comprehensive spatiotemporal single-cell transcriptomic landscape of the colon over a week-long time course of infection. We identified the distal colon as the intestinal segment where most of the host responses were initiated, with distal colonocytes (DCCs) being the most responsive epithelial cells upon the onset of infection. Furthermore, by correlating our findings with human intestinal single-cell transcriptome data, we identified a human colonocyte population that shares many characteristics with murine DCCs. Our study advances the understanding of the cellular and molecular basis of compartmentalized intestinal responses to pathogenic insults and may pave the way for novel preventive and therapeutic strategies to mitigate intestinal damage and combat intestinal infections.

## Introduction

Enteric infections represent a significant global health burden, ranking as the third leading cause of disease-related mortality and accounting for 1.7–2.5 million deaths annually, predominantly among infants and children in developing countries.^1^ Despite the continuous tubular structure of the intestine, it is functionally compartmentalized, with distinct segments exhibiting varying susceptibilities and responses to enteric pathogens.^2^ The progression of an infection, from initial pathogen entry to the immune response and eventual consequences, is largely determined by specific tissues targeted by the pathogen and the localized immune mechanisms deployed to counteract it.^3^ Although covered with a thicker mucus layer (typically a barrier against microbial insults), the colon has been frequently identified as the foremost infected segment for enteric pathogens capable of overcoming the mucin barrier. In particular, the distal colon, the last segment of the intestinal tube, was found to be the primary segment that initiates host inflammatory responses to several pathogens, including *Shigella* species and *C. rodentium*.^4-10^ Besides infection, the distal colon has also been identified as the most affected intestinal segment in several prevalent chronic intestinal inflammatory diseases, especially ulcerative colitis (UC).^11^^,^^12^ Nonetheless, the molecular basis of the immune sensitivity of the distal colon remains elusive.

Among enteric pathogens, *Salmonella* stands out as a particularly prominent threat. It is listed as a high-priority pathogen on the 2024 WHO bacterial priority pathogen list.^13^ Several outbreaks attributed to *Salmonella* serovars are reported annually, with *Salmonella* Typhimurium (S. Tm) being among the most common causal agents.^14^^,^^15^ Clinical observations in humans point to the colon as the primary site of infection, especially in severe or fatal cases.^16-18^ Although systematic information on the lesion distribution in acute human *Salmonella* gastroenteritis is limited, anecdotal evidence consistently associates the most severe pathological changes with the distal colon,^19^^,^^20^ although the biological basis for this site-specific susceptibility remains poorly understood. We speculate that, as stool is temporarily stored in the distal colon prior to defecation (compared to being more mobile in the proximal colon),^21^ there is a greater likelihood of localized microbial growth there.

Susceptible mouse strains, such as C57BL/6 or BALB/c, which carry mutations in the macrophage-encoded *Nramp1* (natural resistance-associated macrophage 1), develop a typhoid fever-like disease upon S. Tm infection. Nonetheless, pretreatment of these mice with broad-spectrum antibiotics renders them susceptible to colitis, closely resembling the inflammatory responses observed in both human colonic infections and animal models of intestinal salmonellosis.^22^^,^^23^ These findings make them a suitable model for investigating the intestinal compartmentalized pathogenesis of *Salmonella*-induced colitis.^24^^,^^25^ Both *in vitro* and *in vivo* studies indicate that S. Tm can interact with various cell types within the intestinal epithelium to establish infection.^20^^,^^26-28^ However, the spatiotemporal dynamics of these interactions in an *in vivo* context remain poorly defined.

To address these gaps, it is crucial to characterize the crosstalk between *Salmonella* and diverse intestinal cell types *in situ* throughout the entire infection course. Recent advances in spatial transcriptomics at the single-cell level have revolutionized our ability to map and spatially localize cell types and states within tissues, disentangling gene expression changes from alterations in cell frequencies.^29^ This technique is particularly powerful for analyzing complex and highly immunologically active intestinal tissue, providing unprecedented insights into cellular subtypes and their interactions during infection.^30-32^

In this study, we present, at single-cell resolution, the spatiotemporal transcriptomic landscape of the colon upon S. Tm infection in an antibiotic-pretreated murine colitis model. Our data offer new insight into the specific contributions of different cell types to both infection and host responses over time, from the initial establishment of the infection until systemic collapse preceding death. By integrating transcriptomic data from the differing but complementary technologies of bulk RNA-seq, single-cell RNA-seq and spatial RNA-seq,^33-36^ we identified previously unrecognized intestinal cellular responses to S. Tm. In particular, we show the prominent responsiveness of distal colonocytes (DCCs) at the initial stages of S. Tm infection, mutans infection highlight the distal colon as a prominent site of the immune response. A comparison with data from the Gut Cell Survey (https://www.gutcellatlas.org/) suggested that a human colonocyte population shares key features with murine DCCs. Our study advances the understanding of the complex response of the intestinal epithelium to pathogenic insults and may pave the way for novel preventive and therapeutic strategies to mitigate intestinal damage and combat intestinal infections.

## Results

### Trajectory of S. Tm infection reflected in the colonic transcriptomic profiles at the bulk, single-cell and spatial levels

To establish an S. Tm-induced colitis murine model, we administered a gavage of 10^9^ CFU of the S. Tm strain IR715^37-39^ to BALB/c mice fed ampicillin-containing (100 µg/mL) water for 3 d. Dissection of infection dynamics revealed that while intestinal infection was detectable 1 d after inoculation, systemic infection became obvious after 3 d, as evidenced both by abundant bacteria in the mesenteric lymph nodes and distal organs, including the spleen and liver, alongside elevated pathological features (Figure S1, A–C). Animals began succumbing to infection from day 4, with every animal (*n* = 23) dying by day 7 (Figure S1D). Our model thus captures the full trajectory of acute enteric infection, from initial colonization to eventual death. Quantification via pathological evaluation and S. Tm distribution across intestinal segments revealed that compared with the small intestine, the cecum and colon exhibited significant higher bacterial loads (Figure S1E,F). Consistent with previous findings,^40^^,^^41^ prominent inflammation was detected in the cecum from the first day post infection. Nevertheless, comparable pathological changes were also detected in the colon at the onset of infection in our model (Figure S1C, E), with infection progressively deepening into the colonic epithelium over time (Figure S1G). Consistent with this, we also observed significant colon shortening starting from 1 d post*-*inoculation (Figure S1H), a hallmark of colonic inflammation. Polymorphonuclear leukocyte (PMN) infiltration and epithelial damage were apparent from day 1, with epithelial damage worsening over time (Figure S1E). Since the cecum pathological mechanisms of S. Tm infection in an antibiotic pretreatment murine model in the cecum have been extensively elucidated in previous studies,^42-45^ our study focused on the non-negligible involvement of colonic segments beyond the cecum.

**Figure 1.**
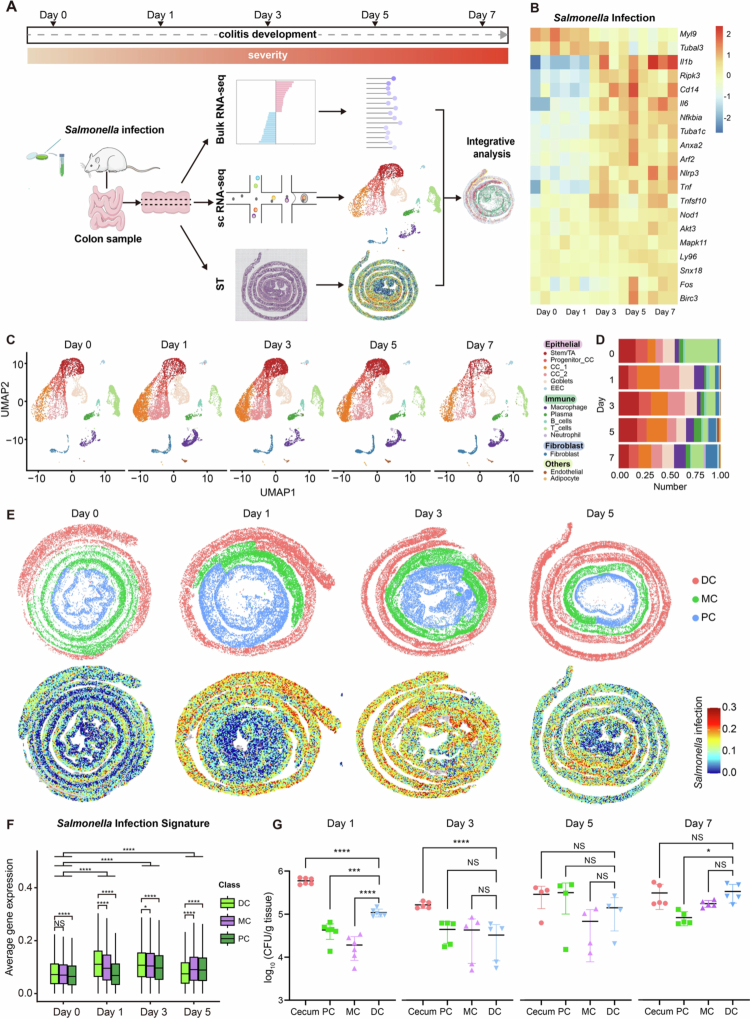
Colonic transcriptomic profiles of the S. Tm infection trajectory reflected at the bulk, single-cell, and spatial levels. (A) Schematic overview of the RNA-sequencing procedure of the colonic tissues from S. Tm-infected mice. (B) Heatmap of the relative expression levels of S. Tm-associated genes over the time course of infection. (C) UMAP visualization of the separated single-cell dataset at different time points during the S. Tm infection course. EEC, enteroendocrine; TA, transit-amplifying cell; CC, colonocyte. (D) Proportion of each cell type at different time points during the S. Tm infection course. (E) Spatial transcriptomic data were divided into distal (DC), middle (MC), and proximal (PC) colon regions (top panel) according to the position of corresponding markers of distal colonocytes, middle colonocytes and proximal colonocytes as established in single-cell transcriptomic analysis (as illustrated in Figure 2G) and projection of a “*Salmonella* infection” gene set (KEGG pathway ID: mmu05132, 129 genes) onto spatial transcriptomic data (bottom panel) at different time points during the course of infection. (F) Box plot of average gene expression of the “*Salmonella* infection” gene set in the proximal (PC), middle (MC), and distal (DC) colonic segments in spatial transcriptome datasets at different time points. (G) Number of invading S. Tm in different colonic segments at different time points over the S. Tm infection course. The Y-axis represents the number of bacteria recovered per gram of tissue. The data are shown as the means ± SEM (*n* = 6 for Day 1, *n* = 5 for Day 3 and 7, and *n* = 4 for Day 5). Box plots show the median, 25th and 75th percentiles, and the whiskers extend to 1.5 × the interquartile range. Significance was calculated by one-way ANOVA with Tukey’s post hoc adjustment; asterisks represent significant differences between the indicated groups. NS, not significant; *p < 0.05, ***p < 0.001, ****p < 0.0001.

To profile the colonic transcriptomic signatures of the S. Tm infection trajectory, colon samples (without cecum) were collected and longitudinally divided into three parts: bulk RNA-seq, single-cell (sc) RNA-seq, and spatial RNA-seq analysis, respectively (Figure 1A). We first used bulk RNA-seq to confirm the existence of S. Tm infection-induced transcriptomic changes, finding that, consistent with expectations, there was significant upregulation of numerous inflammation-related genes, including *IL-1β*, *IL-6*, *Nlrp3*, and *Tnf*, starting from day 3 (Figure 1B, Table S1). Moreover, KEGG enrichment analysis of DEGs (differentially expressed genes) relative to day 0 identified “*Salmonella* infection” (pathway ID: mmu05132, *n* = 129 genes; Table S2) from day 3, which became the most enriched term at day 5 (Figure S2A).

To better characterize the diversity of cell types in the large intestine and their individual responses to *Salmonella* infection, we next performed scRNA-seq analysis on an integrated dataset of 39,706 cells isolated from 10 colonic samples at days 0, 1, 3, 5 and 7 after infection (with 2 parallel samples per time point). No significant batch effects were observed after data integration, as confirmed by the uniform contribution of genes, UMIs, or mitochondrial content per cell (Figure S2B, detailed in Table S3). As expected, the expression of genes associated with the *Salmonella* infection pathway significantly increased over time (Figures S2C and S3A), with strong correlations between the average gene expression across the bulk and scRNA-seq datasets (Figure S4A). Within the scRNA-seq dataset, we identified three major cell populations (epithelial, immune, and fibroblast), which were further clustered into 14 different cell types based on known markers (Figures 1C and S4B). These include five epithelial subsets (expressing *Epcam, Krt8,* and *Krt18*), ordered along the differentiation trajectory from intestinal stem cells to mature colonocytes,^46,47^ fibroblasts (*Col1a1, Col1a2, Adamdec1, Dcn*), endothelial cells (*Cdh5, Cldn5, Pecam1*), B cells and plasma cells (*Cd79a, Cd79b, Cd19, Igha, Jchain, Xbp1*),^47^ T cells (*Cd3d, Cd3e, Cd3g*),^48^ macrophages (*Cd68, Csf1r, Mafb*),^49^^,^^50^ and neutrophils (*Csf3r, Cxcr2*).^51^^,^^52^ Neutrophils, a key indicator of acute bacterial infection,^53^ were negligible before infection but emerged from day 3, accounting for 0.3% of the total cells and increasing to 2% at day 7 (Figure 1C, D). Counts of each cell type at different days of infection are provided in Table S4.

To characterize this transcriptomic landscape spatially, we processed frozen colon sections at days 0, 1, 3 and 5 for spatial transcriptome analysis using the BMK platform (http://www.biomarker.com.cn/zhizao/s1000). After non-coding RNAs (ncRNAs) were filtered out, the resulting dataset comprised 3,406,278 individual spots, which were then segmented into 341,187 cells, with a mean of 85,297 cells per day (range 77,560−99,833) and 338 genes per cell (Table S5). KEGG enrichment analysis of DEGs between time points revealed that the “*Salmonella* infection” pathway was the most enriched term at days 1 and 3 (Figure S3B). Importantly, spatial projection of this gene set demonstrated that the response to *Salmonella* infection was initially localized to the distal colon, gradually spreading to the proximal region at later time points (Figure 1E). Further quantification of the “*Salmonella* infection” gene set in each colonic segment revealed that, along with an overall increase in expression level from day 1, the average expression level of these genes was significantly higher in the distal colon at days 1 and 3 than in the proximal segments. Nonetheless, at day 5, the infection signature distribution reversed, with a higher level in the proximal colon (Figure 1F). To further explore the correlation between the gene expression level and bacterial load in each colonic segment, bacteria invading the different colonic segments were enumerated using a modified tissue-based gentamicin assay. Since gentamicin would kill most bacteria just attached to the tissue, only living bacteria that invade the colonic epithelium could be quantified as CFU by the assay. Consistent with the well-documented primary involvement of the cecum, as supported both by our study and previous studies,^40^^,^^54^ at day 1 post infection, the bacterial load per gram of tissue was higher in the cecum than in other colonic segments (Figure 1G). Nonetheless, it is important to note that, compared with that in the proximal colonic segment (which is anatomically adjacent to the cecum), the bacterial load in the distal colon (anatomically very distant from the cecum) was not only significantly higher but also second only to that in the cecum (Figure 1G). These observations indicate that in addition to the cecum, the distal colon is also a primary target of S. Tm. At day 3, the bacterial load in the cecum significantly decreased, and the difference in bacterial load between the distal, middle and proximal colon became insignificant, echoing the spread of the S. Tm infection signature in the proximal direction and corroborating the results of the spatial transcriptomic analysis (Figure 1E, G). Notably, at the later time points (days 5 and 7), the bacterial load in the distal colon increased to a level comparable to that in the cecum.

Collectively, our colonic transcriptomic profiles at the bulk, single-cell, and spatial levels reflected the S. Tm infection trajectory, providing a reliable foundation for a detailed analysis of the distinct response of individual colonic cell types.

## Colonocytes in distal colon are the most responsive cells at the initial stage of S. Tm infection

**Figure 2.**
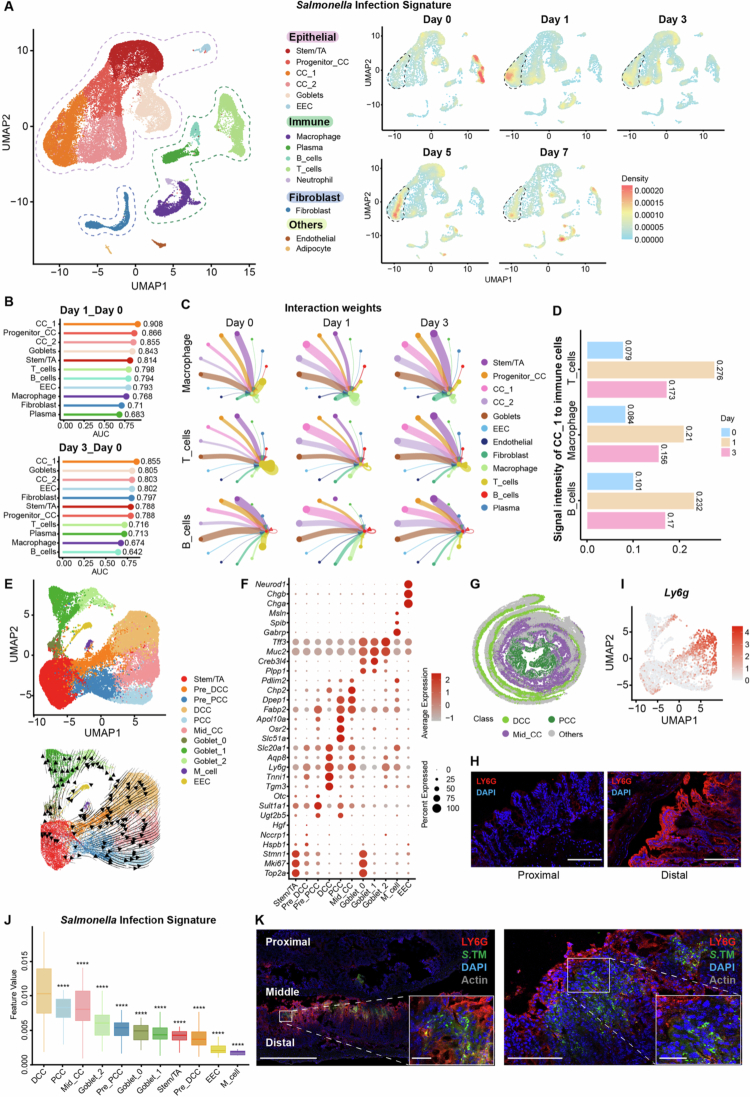
Distal colonocytes exhibit prominent responsiveness at the initial stage of S. Tm infection. (A) UMAP visualization of an integrated single-cell dataset over the S. Tm infection course (*n* = 39,706 cells from 10 samples, 2 replicates at 5 time points) (left panel) and irGSEA scoring of single-cell data using the “*Salmonella* infection” gene set, with the CC_1 cells circled by dashed lines as indicated (right panel). EEC, enteroendocrine; TA, transit-amplifying cell; CC, colonocyte. (B) Lollipop plot of the Augur AUC score for each cell type on days 1 and 3 post S. Tm infection. (C) Weights of interactions between different cell types. The edge width represents the communication probability. (D) Signal strength of CC_1 to immune cells at different time points during the S. Tm infection course. (E) UMAP plot of epithelial cell sub-populations (top panel) with scRNA-seq velocity plots highlighting the differentiation trajectory of intestinal epithelial cells (bottom panel) across all infection time points. (F) Dot plots of the genes used to annotate epithelial cell subpopulations. (G) Spatial mapping of DCCs (dark green), Mid_CCs (purple) and PCCs (light green), with other cells in gray on Day 3. (H) Immunofluorescence analysis showing LY6G protein expression in the proximal and distal colon at Day 0 (uninfected). A representative image is shown (*n* = 3 biological replicates). Scale bars, 100 μm. (I) UMAP visualization of scRNA-seq data reveals *Ly6g* expression at Day 0. (J) Box plot of S. Tm infection signature scoring using “*Salmonella* infection” gene set in different epithelial cell subpopulations at day 1 post infection, as calculated via irGSEA. Box plots show the median and 25th and 75th percentiles, and the whiskers extend to 1.5 × the interquartile range. (K) Immunofluorescence analysis of the colocalization of LY6G and S. Tm in different colonic segments on day 3 post infection. A representative image is shown (*n* = 3 biological replicates). Scale bars, left panel, 500 μm; right panel, 100 μm; insets, 25 μm. Significance was calculated by one-way ANOVA with Tukey’s post hoc adjustment; asterisks represent significant differences compared with DCCs. ****p < 0.0001.

Given the heterogeneous response of intestinal tissues to S. Tm infection, we hypothesized that this variability reflects the distinct roles of different cells and their unique responses (both direct and indirect) to *Salmonella*. To characterize these distinct responses, we projected the “*Salmonella* infection” gene set (Table S2) onto the integrated scRNA-seq UMAP at each of the five time points (Figure 2A). Notably, the most immediate and sustained upregulation of this gene set was in a cluster of differentiated colonocytes (“CC_1”). To quantify the responsiveness of each cell cluster to infection, we used Augur, a tool designed to identify the cell clusters most affected by biological perturbations in single-cell data.^55^ The results demonstrated that the CC_1 cluster exhibited the strongest perturbation at early time points (days 1 and 3) (Figure 2B), although its responsiveness was surpassed by that of other colonocyte clusters by days 5 and 7 (Figure S4C). The immediate responsiveness of the CC_1 cluster to S. Tm infection was further supported by its significantly elevated interaction strength with macrophages, T cells and B cells (Figure 2C, D).

To further annotate the CC_1 cluster in more detail, we reclustered the epithelial cells into 11 subtypes based on previously reported biomarkers^4^ (listed in Table S6) and then estimated the RNA velocity,^4^^,^^56^ (Figure 2E and F). We identified a trajectory of intestinal epithelial cell differentiation from stem cells/transit-amplifying progenitors (TA) to terminally differentiated absorptive colonocytes, goblet cells and enteroendocrine cells (EECs, marked by *Chga*, *Chgb* and *Neurod1*).^48^ Most notably, however, we observed a bifurcation of the differentiated absorptive colonocyte lineages, as reported previously (Figure 2E).^4^ By integrating spatial transcriptomic data, we annotated cell clusters as distal colonocytes (DCCs), proximal colonocytes (PCCs), a transitional ‘middle’ population (mid_CCs), and two clusters of intermediate progenitors (pre_PCCs and pre_DCCs), each with specific marker genes (Figure 2F, G). We found that LY6G, although commonly considered to be a neutrophil marker,^57^ emerged as a marker for DCCs in the single-cell data, as also reported in a previous study.^4^ To validate *Ly6g* expression across different cell types, we conducted a comparative analysis of *Ly6g* expression levels across all the cell types (Figure S5A), which revealed that *Ly6g* presented the highest average expression level in CC_1, which was even higher than that in neutrophils. To further investigate the specificity of *Ly6g* expression, *Ly6g* expression levels across all the epithelial cell subtypes were compared. As shown in Figure S5B, *Ly6g* was significantly enriched in the DCC cluster. Moreover, our immunofluorescence analysis revealed that even before infection, when there were few neutrophils, LY6G was still highly expressed in the epithelial layer of the distal colon, but not the proximal colon (Figure 2H,I). Taken together, these findings suggest that rather than being primarily considered a neutrophil marker, LY6G is also a reliable marker for distal colonocytes.

Using Augur, we assessed the responsiveness of each epithelial subset and found that the LY6G+ pre-DCCs and DCCs subclusters were the most perturbed cell types at days 1 and 3 (Figure S5C), comparable to those of the CC_1 cluster (Figure 2B). We confirmed that the CC_1 cluster comprised LY6G+ cells, supporting its (re)annotation as distal colonocytes (Figure S5D). For each epithelial subset, we examined the expression levels of the “*Salmonella* infection” gene set, finding that after 1 d of infection, there was significantly higher expression of this gene set in DCCs than in all other cell subtypes (Figure 2J and S5E). In addition, the “*Salmonella* infection” gene set was consistently upregulated in DCCs at all the postinfection time points (Figure S5F). Moreover, immunostaining of distal colonocytes with LY6G antibody and S. Tm revealed their colocalization, suggesting the preferential engagement of S. Tm with distal colonocytes (Figure 2K).

## Distal colonocytes exhibit higher responsiveness to stimulation than their proximal counterparts

**Figure 3.**
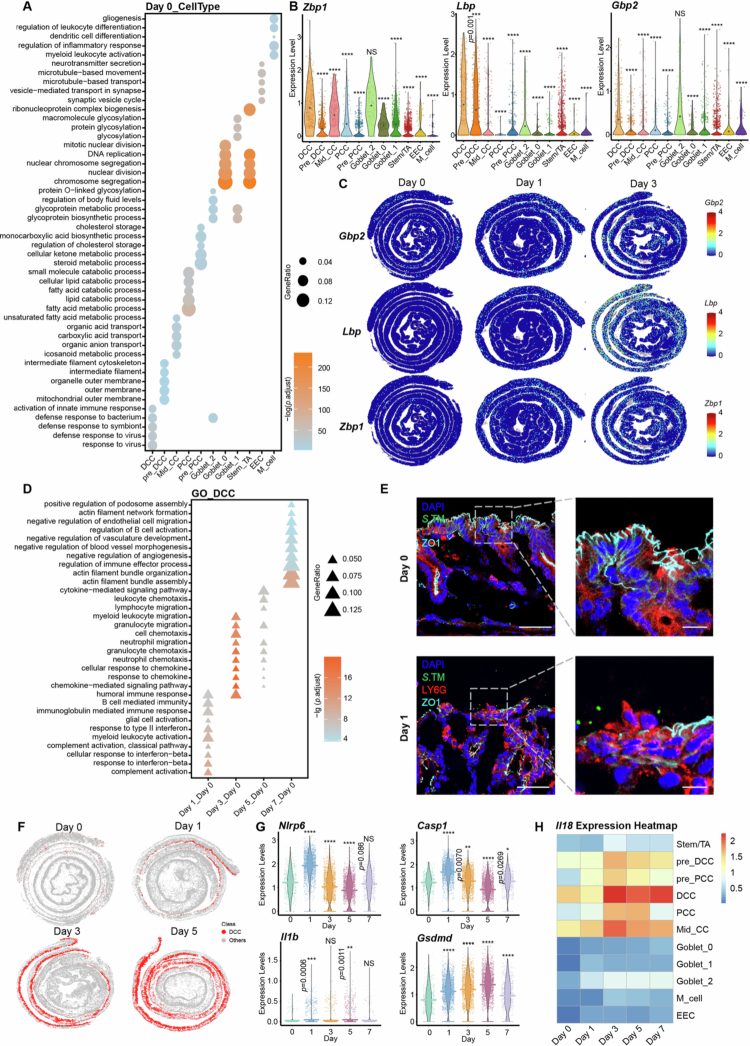
Immunological properties of DCCs contribute to their responsiveness to S. Tm infection. (A) GO enrichment analysis of DEGs in epithelial cell subpopulations in naïve mice. The size of the dots represents the gene ratio of genes enriched in the GO term, and the colors represent -lg (*p*. adjust) value. (B) Violin plots showing the expression of *Zbp1, Lbp,* and *Gbp2* in different epithelial cell type subpopulations in naïve mice. (C) Gene expression of *Zbp1, Lbp,* and *Gbp2* in the spatial transcriptome data at different time points of infection. (D) GO enrichment analysis of DEGs in DCCs at different time points after S. tm infection. The size of the dots represents the gene ratio of genes enriched with the GO term, and the colors represent -lg (*p*. adjust) value. (E) Immunofluorescence analysis of distal colon epithelial barrier damage before infection and on day 1 after S. Tm mutans infection. S. Tm, green; LY6G, red; ZO-1, cyan; and nuclei, blue (DAPI). Scale bars, 50 µm for the left panels, 10 µm for the right panels. (F) Spatial mapping of DCCs at different time points. Red represents DCCs, and gray represents all the other cells. (G) Violin plots showing the expression of *Nlrp6*, *Casp1*, *Il1b* and *Gsdmd* in DCC at different time points during the S. Tm infection course. (H) The heatmap displays *Il18* expression levels across different epithelial cell subtypes at different time points of S. Tm infection. Significance was calculated via one-way ANOVA with Tukey’s post hoc adjustment; asterisks represent significant differences compared with DCCs (B) and Day 0 (G). NS, not significant; *p < 0.05, **p < 0.01, ***p < 0.001, ***p < 0.0001, ****p < 0.0001.

To investigate the reason why DCCs exhibited high responsiveness to S. Tm infection, we performed cell type-specific enrichment analysis of differentially expressed genes (DEGs) among colonic epithelial cell subsets in naïve animals. This revealed that compared with other colonocyte subsets, DCCs were uniquely enriched for immune response-related functions, including ‘activation of the innate immune response’ and “defense response to bacteria” (Figure 3A). In contrast, proximal colonocytes showed no significant enrichment for immune-related functions (Figure 3A, S6A), suggesting that DCCs are intrinsically more sensitive to stimuli (detailed in Table S7). Further examination of the DCC-enriched DEGs revealed that even prior to S. Tm challenge, DCCs expressed high levels of *Zbp1*, a critical signaling initiation factor in the innate immune response (Figure 3B, Table S8). Moreover, LPS-binding factors, including the lipopolysaccharide-binding protein Lbp and guanylate-binding protein Gbp2 – both of which aggregate with LPS, bind to Gram-negative pathogens, and activate pyroptosis – were more highly expressed in DCCs than in other epithelial cell subsets. These factors were further upregulated upon S. Tm infection, as evidenced by both single-cell and spatial transcriptomic data (Figure 3B,C). Similarly, interferon-stimulated factors, including members of the Ifit and Trim families, exhibited DCC-enriched expression in both the single-cell and spatial transcriptomic datasets (Figure S6, B and C).

To explore how DCCs and their progenitors (pre-DCCs) respond to S. Tm infection, we performed enrichment analysis of DEGs at different time points (Figure 3D, S6D, detailed in Table S9). On the first day of post-infection, DCCs exhibited acute innate immune responses (including responses to interferons, complement activation and B-cell activation). By days 3 and 5, these responses shifted towards chemotactic and cytokine-mediated activities (Figure 3D). Consistent with their prominent acute response, the continuity of ZO-1, one of the most important intestinal barrier indicators, was clearly disrupted in the distal colonic epithelium 1 d after infection (Figure 3E). Intriguingly, while pre-DCCs showed alterations similar to those of DCCs at the early stages of infection (days 1 and 3), these progenitor cells also exhibited elevated rates of proliferation and division at later stages, potentially in order to accelerate shedding as an additional mechanism of host resistance. Consistent with this assumption, the velocity length (a proxy for the differentiation rate) of DCCs was markedly accelerated compared to other cell types (Figure S7A, B).^4^ Moreover, using the spatial transcriptomic data, we detected a significant increase in the proportion of DCCs as the infection progressed, further supporting their pivotal role in the host response to S. Tm (Figure 3F and S7C).

To further strengthen the identification of DCCs as key responders, we examined the pathways activated upon S. Tm mutans infection in DCCs via Kyoto Encyclopedia of Genes and Genomes (KEGG) enrichment analysis, and found that several critical PAMP (pathogen-associated pathogen-associated molecular patterns) recognition pathways, including the Nod-like receptor (NLR) signaling pathway, cytosolic DNA-sensing pathway, and Rig-1-like receptor signaling pathway, were significantly upregulated at the initial stage of infection (Figure S6E), along with the upregulation of several critical genes involved in the activation of these pathways, such as *Nlrp6, Casp1, Il1b* and *Gsdmd* (Figure 3G). Immunofluorescence analysis also revealed increased levels of GSDMD (a pyroptosis marker) at the protein level in DCCs after S. Tm mutans infection (Figure S7D). Moreover, we also evaluated alterations in the levels of IL-18, a well-documented inflammatory marker necessary for early epithelial stimulation during S. Tm mutans infection,^58^ both in DCCs and other cell subtypes. While bulk transcriptomic analysis revealed that IL-18 was significantly upregulated upon S. Tm infection in the colonic tissue, further detailed analysis using the single-cell dataset (Figure 3H) revealed that colonocytes (including PCCs, DCCs, mid_CCs and their progenitors) represented the major cell subtypes with significant IL-18 upregulation. More importantly, compared with that in proximal and middle colonocytes, IL-18 expression in DCCs was more pronounced, suggesting that DCCs are the most prominent IL-18 producers upon S. Tm infection. Notably, even before infection, IL-18 expression was higher in DCCs than in other colonocytes.

## Host responses were initially more pronounced in distal colon than proximal region in both infectious and non-infectious colonic inflammation

**Figure 4.**
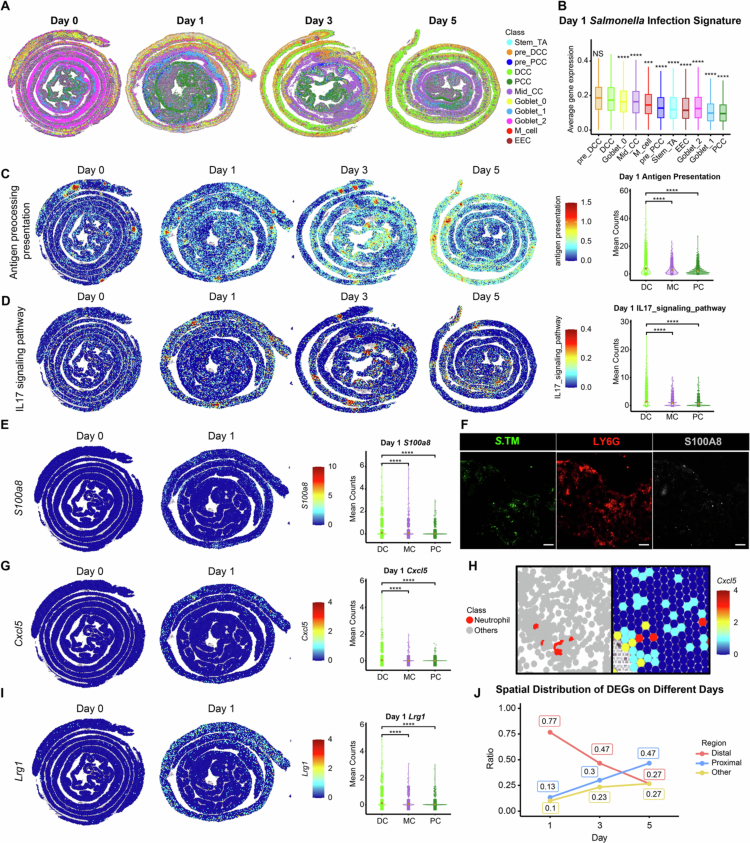
The distal colon is the main intestinal segment that initiates the host response to S. Tm. (A) Spatial mapping of the epithelial cell subpopulations at different time points during the S. Tm infection course. (B) S. Tm infection signature of different epithelial cell subpopulations from the spatial transcriptome data at day 1 after S. Tm infection via the function *AddModuleScore*. (C, D) Spatial mapping of up-regulated “antigen processing and presentation” (C) and “IL-17 signaling” (D) pathways upon S. Tm infection (left panels). Violin plots (right panels) showing the expression of the corresponding gene sets at day 1 in different colonic segments. (E) Spatial mapping of *S100a8* expression in different colonic segments (left panels). Violin plots (right panels) showing the expression of the corresponding genes at day 1 in different colonic segments. (F) Immunofluorescence analysis of S100A8 expression in distal colonocytes on day 3 after S. Tm infection. S. Tm bacteria are green, LY6G bacteria are red, and S100A8 bacteria are gray. Scale bars, 50 μm. (G) Spatial mapping of *Cxcl5* expression in different colonic segments (left panels). Violin plots (right panels) showing the expression of the corresponding genes at day 1 in different colonic segments. (H) Localization alignment of neutrophils and *Cxcl5* gene expression in the spatial transcriptomic data at day 3. (I) Spatial mapping of *Lrg1* expression in different colonic segments (left panels). Violin plots (right panels) showing the expression of the corresponding genes at day 1 in different colonic segments. (J) Proportion of DEGs in specific intestinal regions at different time points after infection. Box plots and violin plots show the median, median and 25th and 75th percentiles, and the whiskers extend to 1.5 × the interquartile range. Significance was calculated by one-way ANOVA with Tukey’s post hoc adjustment, and asterisks represent significant differences compared with DCCs (B) and between the indicated groups (C–E, G, I). NS, not significant; ***p < 0.001. ****p < 0.0001.

To assess the role of DCCs in initiating immune responses following S. Tm infection, we annotated the epithelial cells within the spatial transcriptome dataset using the scRNA-seq annotations (Figure 4A). Projection of the *Salmonella* infection gene set onto the spatial transcriptome data revealed that the pre-DCC and DCC clusters exhibited highest expression levels among all the epithelial cell subsets, further pinpointing their prominent responsiveness to S. Tm infection (Figure 4B). Based on these annotations and their spatial location, we defined the histological upper versus lower segments of the large intestine as the proximal colon, middle colon and distal colon (PC, MC and DC), respectively (Figure 1E). This allowed us to spatially map the infection-related pathways significantly upregulated upon S. Tm infection, as identified in the bulk RNA-seq dataset (Figure S8A). As shown in Figure 4C and D, gene sets of the “antigen processing and presentation” and “IL-17 signaling” pathways in the uninfected colon were primarily localized to mucosa-associated lymphoid tissue (MALT, Figure S8B). However, by 1 d post infection, these pathways become more pronounced in the distal colon than in the proximal colon and spread to the epithelium beyond the MALT. Similar patterns were observed for the gene sets ‘cell adhesion molecules’, ‘Th1 and Th2 cell differentiation’ and ‘TNF signaling pathway’, collectively indicating that the distal colonic segment plays a pivotal role in initiating intestinal immune responses upon S. Tm infection (Figure S9A).

We next analyzed the spatial distribution of the top 50 genes differentially expressed on days 1, 3, and 5 after S. Tm infection identified via bulk RNA-seq analysis. For day 1, 30 of these top 50 genes were identified in the spatial transcriptomic dataset, with 27 showing significant spatial compartmentalization. Among these genes, 23 were predominantly expressed in the distal colon, while only four were localized to the proximal colon (Table S10). Notably, *S100a8*, a prominent marker of inflammatory activity and a well-established predictor of the subsequent course of inflammatory bowel disease,^59^ was among the genes initially upregulated in the distal colon (Figure 4E). A histoimmunofluorescence analysis was concordant with the transcriptomic analysis by showing a significant increase of S100A8 in the distal colon (LY6G-positive segment) upon S. Tm infection (Figure 4F).

In addition, *Cxcl5*, encoding an important neutrophil-attractant chemokine, was disproportionately expressed in the distal colon 1 d post infection (Figure 4G). By day 3, we observed the colocalization of neutrophils with *Cxcl5* (Figure 4H), suggesting that neutrophils also function primarily in the distal colon. This finding is further supported by the higher expression of genes in the “neutrophil extracellular trap formation” KEGG pathway in distal regions than in proximal regions during the initial stages of infection (Figure S9B). Consistent with the transcriptomic analysis, immunofluorescence examination of neutrophil elastase (also known as elastase 2 or ELA2) revealed a significant increase in this neutrophil marker in the distal colonic region (Figure S9C).

Furthermore, several well-known antibacterial proteins and inflammatory factors, including *Lcn2* and *Serpina3n*, were initially induced in the distal rather than the proximal regions (Figure S10A). Similarly, the pyroptotic factor *Gsdmc2* was upregulated in the distal colon 1 d post*-*infection but downregulated at later time points (Figure S10B), suggesting that *Salmonella* infection-induced pyroptosis may first occur in the distal colon, which is consistent with the immunofluorescence analysis validating the elevation of GSDMD at the protein level in DCCs after S. Tm infection (Figure S7D). A tissue-repairing response was also initiated in the distal colon, as evidenced by the upregulation of *Lrg1,* which encodes a leucine-rich *α*-2-glycoprotein involved in colonocyte migration and wound healing,^60^ at day 1 (Figure 4I). In contrast, only four genes (*Reg3g*, *Reg3b*, *Mmp7*, and *Cyp2c55*) were specifically induced in the proximal region of the colon on day 1 (Figure S10C), all of which are antimicrobial peptides known to be expressed predominantly in the small intestine.^61^^,^^62^

To further clarify the spatial dynamics of gene expression over the infection course, we compared the distribution of DEGs across different days. We found that the majority of genes were initially expressed in the distal colon but increasingly shifted to the proximal regions as the infection progressed, suggesting a distal-proximal diffusion pattern of the host response to S. Tm infection in the colon (Figure 4J and S11).

**Figure 5.**
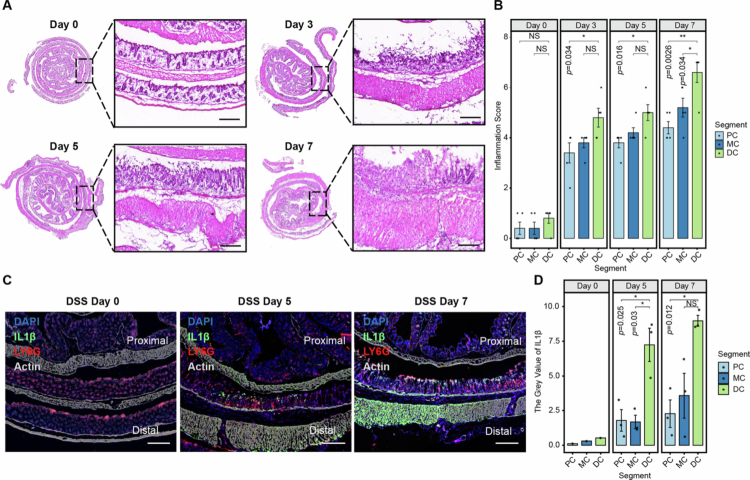
The inflammatory response is more pronounced in the distal colon than in the proximal segments in a murine DSS-induced colitis model. (A, B) Histopathological analysis of different colonic segments at different time points following DSS treatment, as assessed by hematoxylin and eosin (H&E) staining (A, scale bars, 100 μm), and the corresponding histological inflammation scores (B) (*n* = 5). (C, D) Immunofluorescence analysis of IL-1β expression in different colonic segments at various time points after DSS treatment (C, scale bars, 300 μm) and the corresponding quantification of the IL-1β fluorescence intensity over time (D). IL-1β is shown in green, LY6G in red, and F-actin in gray. A representative image is shown (*n* = 3 biological replicates). The data are presented as the mean ± SEM. Statistical significance was calculated by one-way ANOVA followed by Tukey’s post hoc test. Asterisks indicate significant differences between the indicated groups. NS, not significant; *p < 0.05; **p < 0.01.

To determine whether the observed distal colon responses were specific to *Salmonella* infection or reflective of the intrinsic properties of this intestinal segment, we constructed a DSS-induced colitis model in mice ^63^ and analyzed the different colonic segments at different time points. Pathology evaluation via HE staining demonstrated that upon the onset of the DSS-induced colitis, intestinal inflammation and damage were first identified in the distal region of the colon, which extended in a proximal direction. By day 7, when colitis inflammation was full-blown, the pathological score of the distal colon was significantly higher than that of the proximal segments (Figure 5A, B). Moreover, colonic wall thickening, which is primarily attributed to inflammation and immune cell infiltration within the colonic wall, was also found to be more prominent in distal colonic segments than in the proximal colon. Furthermore, examination of IL-1β, one of the most critical pro-inflammatory factors for DSS-induced colitis,^64^ uncovered that IL-1β was predominantly detected in the distal colon both in the mucosal layer and in the thickened submucosal layer (Figure 5C, D). Collectively, these results suggest that inflammation initiation is a reflection of intrinsic immune features of the distal colon, which is consistent with a previous spatial transcriptomic study that used a murine DSS-induced colitis model to identify pronounced changes in human IBD features more in the distal than in the proximal colon.^31^

## A human colonocyte population shares immunological features with murine DCCs

**Figure 6.**
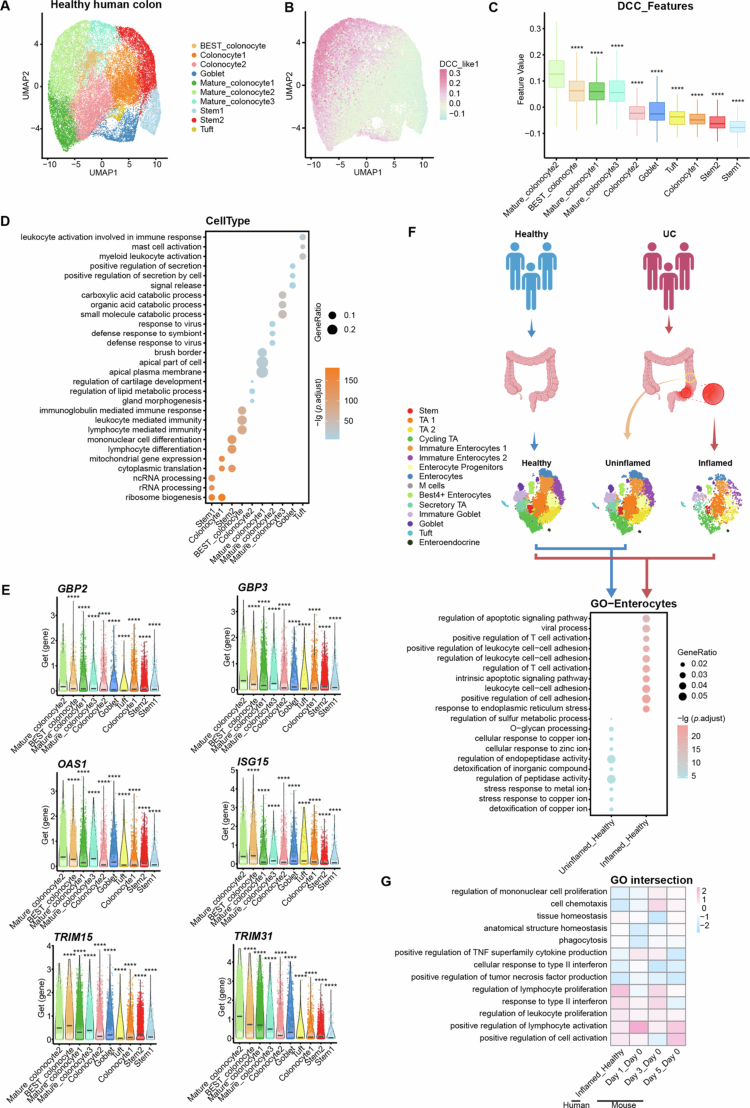
Integration of human datasets revealed shared characteristics between human distal colonocytes and murine DCCs. (A) UMAP visualization of integrated single-cell transcriptomes of epithelial cells from healthy human colon samples extracted from the GCS (Gut Cell Survey) database. (B, C) Seurat *AddModuleScore* evaluation of human healthy colon epithelial cell subpopulations using murine DCC DEGs (that is, genes differentially expressed in DCCs on the basis of adjusted *p*-value < 0.05 and log_2_FC > 1). The color intensity in the UMAP plot (B) represents the relative expression levels of the gene set in each cell subpopulation. The detailed feature values are shown in panel (C). (D) GO enrichment analysis of DEGs between different epithelial cell subpopulations in healthy human colon samples. The size of the dot represents the gene ratio of genes, and the color represents the -log (*p*.adjust) value. (E) Violin plots showing the expression of *GBP2, GBP3, OAS1, ISG15, TRIM15, and TRIM31* in different epithelial cells. (F) GO enrichment analysis of DEGs of putative human DCCs from the comparison between the UC groups (uninflamed, inflamed) and the healthy control group. The size of the dot represents the gene ratio of genes, and the color represents the -log (*p*.adjust) value. (G) Intersection of enriched GO terms in human DCCs between the inflamed UC group and the healthy control group and enriched GO terms in murine DCCs at different time points after S. Tm. Box plots show the median, 25th and 75th percentiles, and whiskers extend to 1.5 × the interquartile range. Significance was calculated by one-way ANOVA with Tukey’s post hoc adjustment, and asterisks represent significant differences compared with the mature colonocyte2 (putative human DCC) cluster. ****p < 0.0001.

To explore the translational potential of our findings in murine DCCs, we investigated whether the murine DCCs identified in our study have human counterparts. To do so, we leveraged single-cell transcriptomic data from the Gut Cell Atlas (https://www.gutcellatlas.org/), extracting 36,390 single-cell transcriptomes of colon tissues from 30 healthy individuals. These data were integrated and annotated into five cell populations based on established markers ^48^ (Figure S12A, B). The epithelial cells were further reclustered into 10 subclusters (Figure 6A and S12C), which were annotated using biomarkers from the original studies (Table S6). Among these, three epithelial subsets were identified as mature colonocytes, which were further distinguished using murine DCC features (that is, DEGs in DCCs compared with the other epithelial subsets in this study; Figure 6B). Notably, one cluster of mature human colonocytes (‘Mature_colonocyte2’) exhibited characteristics resembling murine DCCs, with markedly higher average expression levels of their respective orthologs (Figure 6C).

To characterize the functions of these putative human DCCs, we performed GO enrichment analysis on genes differentially expressed in the human colonic epithelial cell subset. As shown in (Figure 6D), human DCCs demonstrated several immune response-related functions, including “defense response to virus” and “defense response to symbiont”, mirroring the functional profile of murine DCCs. This finding suggests that, like their murine counterparts, human DCCs are likely more sensitive to external microbial stimuli. Further examination of human DCC-enriched DEGs revealed several genes potentially underlying this phenotype (Figure 6E and S12D), including LPS-binding GBP family members (*GBP2* and *GBP3*), and interferon-stimulated factors, including *OAS* and both the *ISG15* and *TRIM* families. These findings reinforce the notion that human DCCs share common immunological genetic features with murine DCCs.

To evaluate the responsiveness of putative human DCCs in intestinal inflammation, we analyzed publicly available single-cell transcriptome data collected from patients with ulcerative colitis (UC), a prevalent bowel inflammatory disease in which the distal colon is often the most affected intestinal segment.^65^ Data from UC patients included inflamed colonic tissues, adjacent normal uninflamed tissues, and healthy controls.^65^ We scored the cell clusters annotated in the original study using murine DCCs gene set and identified a cluster of cells, “enterocytes”, with the highest proportion of DCC features (Figure S12E, F), which we thus defined as human DCCs. Intergroup comparison and functional enrichment analysis revealed that human DCCs from inflamed UC tissues presented upregulated pathways related to immune cell activation and leukocyte cell adhesion compared to healthy controls. In contrast, DCCs from the uninflamed tissue of UC patients show no significant immune-activating alterations relative to those from healthy controls (Figure 6F), indicating that human DCCs actively participate in bowel inflammation. More importantly, immune-activating responses of human DCCs in UC inflammation, such as lymphocyte activation, leukocyte proliferation, and response to type 2 interferon, were also observed in murine DCCs upon S. Tm infection (Figure 6G and S12G). This finding suggests conserved functional roles for DCCs across species in mediating immune responses to intestinal inflammation.

## Discussion

The intestine is a highly compartmentalized organ with unique characteristics and functions for different segments^2,66^, which are crucial for maintaining homeostasis and allowing the immune system to respond appropriately to diverse challenges within the gut. Nonetheless, while the small intestine is well known for its regional compartmentalization, the molecular regionalization of the colon and its implications for colonic diseases remain less clear.^2^ By using an S. Tm colitis model in antibiotic-treated BALB/c mice (which closely resembles the responses observed in the human colon for intestinal salmonellosis^40^), we present a comprehensive spatiotemporal single-cell transcriptomic landscape of the colon over the entire S. Tm infection course. Our data highlight the longstanding conundrum of the intestinal segment primarily affected by S. Tm, drawing particular attention to the role of the distal colon, where most of the host responses to S. Tm infection appear to be initiated. Furthermore, a cell type-specific analysis of DEGs suggests that distal colonocytes are intrinsically more sensitive and reactive to external microbial stimuli than their counterparts in the proximal colon, as evidenced by higher expression of immune sensors, LPS-binding factors and interferon signaling components.

Efforts to develop reliable animal models that recapitulate the human colitis pathology induced by *Salmonella* have yielded valuable insights.^26^^,^^67^ For instance, rhesus monkeys developed salmonellosis symptoms similar to those of humans following oral administration of the pathogen, with infiltration of PMNs into the mucosa and sloughing of epithelial cells.^28,68^ In *Nramp*-mutated susceptible mouse strains, natural S. Tm infection causes typhoid-like disease, whereas streptomycin pretreatment makes these mice susceptible to *Salmonella* colitis, mimicking human colon inflammation.^25^^,^^40^ Accordingly, *Salmonella* pathogenicity and the molecular basis of host defense have been intensively studied.^27^^,^^67^ Nevertheless, the cellular and molecular basis of *in vivo* compartmentalization of *Salmonella*-induced colitis and the precise mechanisms of *Salmonella*-host interaction at early infection time points remain unclear, as a comprehensive spatiotemporal landscape of the molecular dynamics of infection is lacking. In previous studies, the cecum has been extensively documented for its prominent inflammatory response to S. Tm mutans infection^42^^,^^54^^,^^69-73^ since the bacteria were first detected in the cecum. However, the initial detectable colonization site did not necessarily indicate the ultimate tissue tropism. Although *C. rodentium* has been well documented as a pathogen exhibiting clear tropism to the distal colon,^4^ and its primary colonization of the mouse was found to take place within the cecum.^6^ As such, in studies that focused on the caecal response to S. Tm infection,^40^^,^^41^ significant infection symptoms in other colon segments beyond the caecum were consistently detected, although the mechanistic details of colonic involvement remain to be systematically elucidated. Consistently, in our model, in addition to the cecum, comparable pathological changes were also detected in the colon upon the onset of infection, indicating that the colon was also among the primary targets of S. Tm. In particular, rather than the proximal colonic segment (which is anatomically adjacent to the cecum), our analysis revealed that the host responses in the distal colon were more pronounced than those in its proximal counterpart, especially at the initial stage of infection. These results echo anecdotal clinical observations in human patients that the most severe pathological changes during S. Tm infection involve the distal colon.^74^ For example, necropsy findings from six patients who died of *Salmonella typhimurium* infection revealed severe colitis, with the distal colon and rectum most severely affected.^74^ Subsequent clinical studies have corroborated these findings.^17-19^^,^^75^

Recent advances in single-cell and spatial transcriptomics have provided us with a new opportunity to investigate the cellular and molecular basis of the regionalization of enteric diseases at an unprecedentedly high resolution.^4^^,^^31^ Advanced by an integrated analysis of both single-cell transcriptomic and spatial transcriptomic data, our study presented a comprehensive spatiotemporal landscape of S. Tm-induced colitis and characterized the differential involvement of different colonic segments, especially at early time points after S. Tm infection. Our results emphasize the prominent involvement of distal colonocytes with S. Tm infection and its pivotal role in initiating host responses. While DCCs have been demonstrated to play essential roles in activating the adaptive immune response after *C. rodentium* infection by orchestrating the T cell response via IL-22 signaling,^4^ our study reports new insights into the immunological properties of naive-state DCCs in comparison with those of other colonocytes, which may endow them with relatively greater sensitivity and reactivity to external stimuli than their proximal counterparts. By illustrating the molecular basis of the prominent responses of DCCs at the early stage of S. Tm mutans infection, our study provides a new perspective on understanding the role of DCCs in activating innate immune responses to pathogenic insult. Supporting this, among all the epithelial cell subtypes, DCCs showed the highest expression of *Zbp1*, a well-documented innate immune sensor,^76^ in addition to their higher expression of LPS-binding factors and a set of interferon-stimulated factors. Nonetheless, it should be mentioned that the susceptibility of the distal colon was not specific to S. Tm since other critical enteropathogenic pathogens, including *Shigella* spp. and *C. rodentium*, were also found to exhibit clear tissue tropism to the distal colon.^4^^,^^10^^,^^77^ To further support the prominent responsiveness of the DCCs, we provided additional spatial transcriptomic evidence at multiple time points to strengthen the conclusion that DCCs play a pivotal role in triggering host responses to bacterial insults by demonstrating that most of the upregulated infection-related pathways, as well as the top 50 DEGs after S. Tm mutans infection, were first positioned in the distal colon, and then spread in a proximal direction at later time points.

Although acute bacterial infection differs fundamentally from inflammatory bowel disease, a number of previous clinical studies have provided evidence showing that *Salmonella* colitis in humans may present as a segmental colitis resembling pathological changes in idiopathic ulcerative colitis and Crohn’s disease. These previous studies revealed that despite the significant differences in causes, some inflammatory responses are shared between *Salmonella*-induced colitis and UC.^78-80^ Our study implies the real possibility that DCCs, in both mice and humans, may serve as important danger-sensing and response-initiating cells in the intestine, which would certainly contribute to the frequent involvement of the distal colon in inflamed intestinal diseases with both infectious and noninfectious causes. Besides being targeted by the pathogens, the distal colon was also demonstrated to be the most affected intestinal segment in several prevalent intestinal inflammatory diseases, especially ulcerative colitis (UC).^81^^,^^82^ UC is characterized by relapsing and remitting mucosal inflammation, starting in the distal colon and extending in a proximal direction.^82^ Sufficient colonoscopic evidence suggests that the distal end of the lesion has the most abundant pathological features,^83^ although the exact cause of regionalization of UC is still elusive. Interestingly, the susceptibility of the distal colon to inflammation is conserved between mice and humans, as revealed by a spatial transcriptomic study in a murine DSS-induced colitis model.^31^ By integrating murine colitis spatial transcriptomic data with human fetal gut single-cell transcriptome data and inflammatory bowel disease (IBD) patient bulk transcriptome data, dramatic changes in IBD features were identified in the distal rather than the proximal colon, in agreement with the phenotypes observed in UC patients.^78^ Nonetheless, the cell subtypes responsible for higher levels of damage/inflammation in the distal colon were not thoroughly investigated in this study. By revealing the sentinel immune functions of distal colonocytes and uncovering their functional conservation between mice and humans, our study provides a deeper understanding of the cellular and molecular basis of the predominant involvement of the distal colon in intestinal inflammation, which may help with pathogenesis research and therapeutic development for UC and other inflammatory bowel diseases in humans.

Nevertheless, several limitations of the current study should be clarified. First, despite being a versatile murine model to study *Salmonella* pathogenesis, nonphysiological elimination of the microbiota by antibiotic pretreatment may undermine the model’s ability to reflect natural colonization dynamics and could complicate pathogen‒commensal interactions. The possible roles of microbiota disruption and a compromised epithelial barrier in shaping DCC responsiveness and infection trajectories should be addressed in future studies. Second, only female mice were used in our study in order to facilitate comparisons with previous studies.^84-87^ The inclusion of both sexes would provide a more comprehensive understanding of host responses and allow exploration of potential sex-specific effects. Third, most of the findings on the roles of DCCs in initiating inflammation are based on transcriptomic data analysis with corroboration using histoimmunofluorescence, and so further mechanistic experiments are necessary to establish an evidential basis for causality. Finally, in addition to the significant increase in neutrophils, fluctuating counts and differential spatial distributions of other cell populations (including immune and goblet cells) were also apparent over the course of infection in our transcriptomic analysis, which may open up further lines of inquiry.

In summary, we present a spatiotemporal landscape of the cellular and molecular hierarchy of *Salmonella*-induced colitis in mice, offering insight into the molecular basis by which intestinal infection and inflammation are compartmentalized and paving the way toward preventive and therapeutic strategies for mitigating intestinal infections.

## Materials and methods

### Ethical approval

The study was conducted in compliance with the appropriate ethical guidelines and regulations. The animal studies were approved by the Biomedical Ethics Committee of the Health Science Center of Xi’an Jiaotong University (ethical approval No. XJTUAE2025-53).

### Reagents

The commercial reagents used in this study are listed in Supplementary Table 11.

### S. Tm infection procedure

Six- to eight-week-old BALB/c female pathogen-free mice were procured from Beijing KeAo Biological Co., Ltd. All relevant ethical regulations were strictly adhered to. During the experiment, healthy BALB/c mice were randomly allocated to each group. The mice were housed in colony cages within a pathogen-free environment, with the temperature maintained at 21−23 °C, relative humidity at 50%−60%, and a 12 h light/12 h dark cycle. All the mice were fed ad libitum with a standard chow diet. Prior to bacterial inoculation, the mice were pretreated with 100 µg/mL ampicillin in distilled water for 3 consecutive days. For the survival curve experiment, a total of 46 mice were used, with 23 in the experimental group and 23 in the control group. For the detection of S. Tm dissemination, a total of 42 mice were divided into 4 groups (d1, 2, 3, 4, 5, 6 and 7). Each group included 2 control mice and 4 experimental mice. S. Tm strain *IR715*,^37-39^ which harbors a GFPuv plasmid conferring ampicillin resistance, was cultured overnight in TSB medium containing ampicillin (100 µg/mL) and then subcultured to the early exponential phase with an optical density of 600 nm, which reached 0.4–0.6. The bacteria were then collected by centrifugation and resuspended in warm sterile PBS. The mice were inoculated with 10^9^ CFU in a total volume of 200  µL of PBS by gavage. At the end of each experiment, the mice were sacrificed by cervical dislocation. Immediately after sacrifice, the colon, mesenteric lymph nodes (MLNs), liver, and spleen were carefully excised for subsequent analysis. The MLN, liver, and spleen tissues were harvested and processed to quantify the colony-forming units (CFUs). The colon was harvested and fixed in 4% PFA for tissue staining and immunofluorescence.

### Colonic tissue-based gentamicin protection assay

BalB/c mice were killed post infection. The colon was excised and rinsed thoroughly with sterile PBS containing 100 μg/mL gentamicin, followed by 3 additional rinses. Then, the colon was subsequently divided into the cecum, proximal colon, middle colon, and distal colon as described previously.^88^ Subsequently, different intestinal segments were weighed and incubated in PBS/100 μg/mL Gent for 30–60 min. Before plating, the tissues were washed 6 times with sterile PBS. Finally, the number of bacteria per gram of tissue was determined.

### DSS colitis model

Six- to eight-week-old BALB/c female pathogen-free mice were used to establish a dextran sodium sulfate (DSS, MW = 40 kDa; Yeasen, Shanghai, China)-induced colitis model. The mice were divided into five groups: a control group (day 0, *n* = 5) and DSS treatment groups (day 1, *n* = 5; day 3, *n* = 5; day 5, *n* = 5; day 7, *n* = 5), with a total of 25 mice. Specifically, the mice in the DSS treatment groups were provided with drinking water containing 3% (w/v) DSS ad libitum until the day of sacrifice, while the control group received normal drinking water. General health and body weight were monitored daily. The mice in each group were sacrificed on days 0, 1, 3, 5, and 7, and their colon tissues were collected for histological and immunofluorescence analysis.

### Immunostaining and confocal microscopy

The infected colonic tissues were embedded in optimal cutting temperature (OCT) compound and cut into 10 μm thick sections. The OCT sections were first fixed with 4% paraformaldehyde at room temperature for 15 min, followed by permeabilization with 0.3% Triton X-100 in PBS for 10 min at room temperature and blocking in 3% BSA in PBS for 1 h. The sections were then incubated with fluorescein-conjugated primary antibodies overnight at 4 °C. After thorough washing in PBS-T (PBS containing 0.1% Tween−20), sections were further stained with Alexa Fluor 647- or TRITC-conjugated phalloidin (Yeasen, China) for 30 min and mounted with DAPI-containing Fluoromount-GTM reagent (Yeasen, China) onto glass slides and imaged as described below. The tissue samples were imaged using a Leica STELLARIS 5 laser scanning confocal microscope. Scale bars were added by means of the Leica Application Suite X (LAS X) software. Image quantification and channel merging were performed using ImageJ v2.1.0^89^ software.

In order to quantify the bacterial load in different intestinal segments after infection, the tissue sections were subjected to immunofluorescent staining with a FITC-conjugated anti-S. Tm LPS antibody. Fluorescence images were acquired with the same exposure settings. The fluorescence signals from each experimental group (different days post infection) were normalized against those from the uninfected controls.

### S. Tm tracking and invasion depth quantification

To quantitatively analyze the depth of S. Tm invasion into the colonic epithelium, confocal immunofluorescence images were acquired at different time points post infection (Days 1, 3, 5, and 7). Automatic segmentation of individual S. Tm values was performed using the TrackMate plug, which provides their x and y coordinates. The positions of the intestinal epithelial layer (represented as a solid line) and the basal layer (represented as segmented lines) were manually annotated using Fiji ImageJ v2.1.0^89^ (Figure S1G, right panel). The coordinates of these intestinal boundaries were subsequently extracted using GetData Digitizer 2.22. Finally, MATLAB R2023a was utilized to map the positions of S. Tm onto the layers and to calculate the depth of S. Tm invasion.

### Histopathology

The OCT-embedded (Sakura) sections were cut into 10-µm-thick slices and stained with hematoxylin and eosin (H&E), as previously described.^90^ The images were captured with a slide scanning microscope (Pannoramic 250 FLASH III 3.0.0, 3DHISTECH). The histopathology score was determined blindly as previously described.^40^

### Transcriptomic analysis of a mouse colon sample

Transcriptome sequencing (bulk RNA-seq) was performed on the colons of BALB/c mice on different days after S. Tm gavage (days 0, 1, 3, 5 and 7; *n* = 3 at each time point). The colons were washed 5 times with PBS and preserved in RNALater (Beyotime) at –80°C until further use. The RNA extraction, RNA library preparation and sequencing were carried out by Novogene (Shanghai, China). Briefly, sequencing libraries were generated using the NEBNext Ultra RNA Library Prep Kit for Illumina (New England BioLabs, USA), with index-coded samples clustered according to the manufacturer’s instructions using a cBot Cluster Generation System and the TruSeq PE Cluster Kit v3-cBot-HS (Illumina). Libraries were sequenced using an Illumina NovaSeq 6000 to produce 150 bp paired-end reads. The raw reads were cleaned using fastp v0.19.7^91^ with the parameters -g -q 5 -u 50 -*n* 15 -l 150. The cleaned reads were then aligned to the mouse reference genome GRCm39 (downloaded from Ensembl v113) using STAR v2.7.9a^92^ with default parameters. The resulting BAM files were coordinate-sorted using samtools v1.13,^93^ and then analyzed using featureCounts v2.0.0^94^ with the corresponding gene annotation files (https://ftp.ensembl.org/pub/release−113/gff3/mus_musculus/Mus_musculus.GRCm39.113.gff3.gz). The gene expression matrix was generated from this analysis. Differential expression analysis was performed on the expression matrix using the DESeq2 v1.32.0 package,^95^ and the “counts” function was used to obtain the normalized data. Differentially expressed genes were identified with thresholds of adjusted *p*-value < 0.05 and |log_2_FC| > 1.

### scRNA-seq and data analysis

#### scRNA-seq library preparation and sequencing

Colonous tissues were collected from mice on five different days (Days 0, 1, 3, 5, and 7) before and after the intragastric administration of S. Tm. Each sample was processed within 30 min of collection. The collected tissues were gently cleaned, cut into pieces of approx. 3–5 mm,^3^ and incubated on ice at 4 °C. The filtrates were precooled to 4 °C with PBS and allowed to settle for 10–50 s before discarding the supernatant. Epithelial cells and some of the lamina propria cells were collected by gentle dissociation using a Lamina Propria Dissociation Kit (mouse, #130-097-410; Miltenyi Biotec) for 15 min. The remaining tissue was then subjected to further harsher dissociation using 500 μg/mL type VIII collagenase (Sigma-Aldrich) and 20 μg/mL DNase I (Roche) on a shaker for another 15 min. During digestion, the tissues were pipetted vigorously every 7 min to facilitate cell release. After 15 min, the cell suspension was filtered, counted, and quality-checked. The cells collected from these two steps were combined for the assessment of cell viability. The cells were then diluted to 5 × 10⁵–1 × 10⁷ cells/mL, followed by staining with the Live/Dead Cell Staining Kit (YB140632-1000, Shanghai Yubo Biotechnology Co., Ltd.). The stained cell suspension was mounted to a Countstar cell counting chamber and analyzed using a Countstar Rigel S2 fully automated cell fluorescence analyzer to evaluate cell viability. Dead cells were removed with a Dead Cell Removal Kit (Miltenyi Biotec, USA), with the cell survival rate per sample generally above 87%. The data quality of single cells before sequencing are detailed in Table S12. scRNA-seq library preparation and sequencing were then performed by Genergy Bio (Shanghai, China) using the 10x Chromium single-cell platform with version 3 reaction chemistry, in accordance with the manufacturer’s instructions. Single-cell suspensions were prepared as barcoded scRNA-seq libraries using a Chromium Single Cell 3’ Reagent Kit v3 (10x Genomics, Shanghai, China), with libraries sequenced on an Illumina NovaSeq 6000 (Illumina, San Diego, USA).

#### Single-cell data processing

The single-cell RNA-seq processing and sample-integration workflow were lightly adapted from those previously described.^96^ In brief, cell x gene count matrices were produced for each sample using the Kallisto/bustools (KB) v0.28.2^97^ ‘count’ workflow with the parameter ‘--filter bustools’, building its transcriptomic index from the mouse genome GRCm39 (downloaded from Ensembl v111). For each sample, we retained only those cells with > 1000 genes, > 2000 UMIs and where < 10% of the total reads were from mitochondrial genes, and only those genes detected (i.e., having one or more reads) in at least 3 cells. Each count matrix was processed using Seurat v5.0.1^98^ with the SCTransform normalization workflow and the ‘glmGamPoi’ method (implemented with the R packages sctransform v0.4.1^99^ and glmGamPoi v1.14.3,^100^ respectively), setting the mitochondrial gene content, total gene count and UMI count, and both the ‘S score’ and ‘G2M score’ as regression variables (using the set of 97 human cell cycle phase-associated genes included in Seurat as the ‘cc.genes.updated.2019’ variable, which was programmatically converted to their corresponding one-to-one mouse orthologue using the R package gprofiler2 v0.2.3^101^). Doublets were then predicted using DoubletFinder v2.0.4^102^ with a set clustering resolution for modeling homotypic doublets (0.3) and a set proportion of expected doublets (7.5%) per sample; only cell barcodes classified as ‘singlets’ were retained for further analysis.

Finally, all 10 samples were integrated using Seurat’s CCA (canonical correlation analysis) method with 5,000 integration features. To interpret the structure of the integrated dataset, transformed gene counts were used for principal component (PC) analysis, with elbow plots used to define an optimal number of PCs for downstream analysis, including dimensional reduction and visualization with the UMAP method. As described previously,^96^ the elbow was programmatically determined as the PC where the percentage change in variation between it and the next PC < 0.1%, or where all previous PCs had cumulatively accounted for >90% of the total variation and the current PC only accounts for <5%, whichever was lower. We retained all PCs up to and including the elbow PC. These PCs were used to construct a k-nearest neighbor graph, which we clustered using the Smart Local Moving algorithm with a resolution of 0.2 (empirically selected on the basis of both manual review and a Clustree v0.5.1^103^ dendrogram). To subcluster the epithelial cells, the corresponding cells were extracted, and the above steps (normalization, dimensionality reduction, and clustering) were repeated. To annotate clusters in an unbiased manner, we performed an all-against-all differential expression analysis using Seurat’s FindAllMarkers function with the parameters min.pct = 0.25, logfc.threshold = 0.25 and only.pos = TRUE. These parameters require that a gene is expressed in >25% of the cells in each cluster and to have at least 1-fold higher average expression in a given cluster relative to all other clusters.

#### Identification of differentially expressed genes.

Pairwise differential gene expression analysis (of one cluster against another named cluster) was conducted using the *FindMarkers* function in Seurat with parameters min.pct = 0.1 and only.pos = TRUE. We implemented the Wilcoxon rank-sum test and, to consider the result significant, also required a minimum log_2_FC threshold of 1 and an adjusted *p*-value < 0.05.

#### Single-cell RNA-seq data analysis

Module scores of the expression level of gene sets or genes were calculated using the *AddModuleScore* function implemented in the Seurat package. To assess differences in gene set activity across each cell type, the irGSEA v3.2.9 package^102^ was used to calculate gene set enrichment scores. The AddModuleScore and irGSEA scores for different cell types were visualized using the R package ggplot2 v3.5.1.^101^ The R package Augur v1.0.3^55^ was used to perform cell-type prioritization and generate the uniform manifold approximation and projection (UMAP) plot of the area under the curve (AUC) for cluster responsiveness to S. Tm.

#### *Salmonella* infection gene set

To validate *Salmonella* infection across different datasets, bulk transcriptome data were subjected to an analysis of differentially expressed genes (DEGs) between the control group and each postinfection time point (day 1 vs. day 0, day 3 vs. day 0, day 5 vs. day 0, and day 7 vs. day 0). DEGs that exhibited significant changes at different time points (adjusted *p*-value < 0.05) were identified. Subsequently, the Kyoto Encyclopedia of Genes and Genomes (KEGG) functional enrichment analysis was carried out on these significant DEGs for each day. Finally, the term “*Salmonella* infection” was extracted from the KEGG results of different days, and all genes associated with this term were defined as the characteristic gene set for “*Salmonella* infection” (see Supplementary Table S2 for details).

### Enrichment analysis

The clusterProfiler v4.6.2^104^ R package was employed for gene set enrichment. The gene sets used were Gene Ontology (GO) biological process gene sets. The input for GO enrichment analysis was a set of gene signatures specific to clustered cell types obtained from Seurat (adjusted *p*-value < 0.05 and log_2_FC > 1). Concurrently, for KEGG enrichment analysis, the input was a set of gene signatures representing different days post infection in comparison with the control group. These gene signatures were sourced from bulk RNA-seq, single-cell RNA-seq (scRNA-seq) and spatial RNA-seq (stRNA-seq) data. In bulk RNA-seq analysis, significant genes were defined as those satisfying either of the following conditions: an adjusted *p*-value < 0.05 and |log_2_FC| > 1, or an adjusted *p*-value < 0.05 and log_2_FC > 1. For scRNA-seq and stRNA-seq analysis, significant genes were those with an adjusted *p*-value < 0.05 and an average |log_2_FC| > 1.

### Cell‒cell interaction analysis

To analyze intercellular communication among different cell types, the R package CellChat v1.6.1^105^ was utilized. The input data consisted of the normalized counts obtained from Seurat, and standard preprocessing functions, namely, “identifyOverExpressedGenes” and “identifyOverExpressedInteractions”, were employed. The core functions “computeCommunProb” and “aggregateNet” were applied with standard parameters. The “netVisual_circle”^56^ function was subsequently used to visualize the interaction intensity targeting specific cell types.

### RNA velocity analysis

RNA velocity analysis was carried out using the scVelo package v0.2.2^56^ in Python v3.8.5. To perform the analysis, the unspliced and spliced variant count matrices were first obtained using the 10 × pipeline implemented in velocyto v0.17.16.^106^ These were then combined with an AnnData object containing the UMAP information and cluster identities determined in the Seurat analysis. The combined dataset was then processed using the scVelo pipeline, with the per-gene ratio of unspliced:spliced RNA normalized and filtered using the default settings. Afterward, the first and second moments were calculated for velocity estimation. Following the moment calculation, the dynamic model was used to calculate the RNA velocities. The RNA velocities of each epithelial cell in all samples were calculated and projected onto the pre-existing UMAP embedding using the ‘velocity_embedding_stream’ command.

### Tissue processing and spatial transcriptomics sequencing

OCT-embedded colonic tissues were cut into sections with a thickness of 10 µm and placed onto chilled tissue optimization slides and gene expression slides (BMKMANU S1000). The gene expression slides were fixed and stained with hematoxylin and eosin (H&E) and imaged using a Panoramic MIDI microscope at 40X magnification. Nuclei were also stained and imaged for subsequent cell segmentation. After reverse transcription and spatial library construction, the library was sent for quality control and sequenced on the Illumina NovaSeq X Plus platform to generate 150 bp paired-end reads.

### Spatial transcriptome data preprocessing

#### Raw data processing for bin generation

For each sample, FASTQ files and manually aligned histology images were analyzed with BSTMatrix v2.3. The reads were mapped to the mouse reference genome GRCm39 using STAR v.2.7.9a.^92^ The processed data were imported into R using Seurat v5.0.1.^98^ Spatial spots featuring more than 30% of the mitochondrial genes and fewer than 300 genes were filtered out. Genes with counts in fewer than 5 spatial spots were discarded. The raw counts were normalized with the SCTransform function of Seurat using the ‘‘assay = spatial’’ parameter. Based on the results of the raw data processing, the original spots were resegmented into new bins (61 original spots were merged into one) to obtain sufficient gene expression data. Finally, level 5 (50 μm) matrix data were obtained for spatial transcriptome scoring.

#### Raw data processing for cell segmentation

Raw sequencing files were processed using BSTMatrix v2.3 to conduct cell segmentation and obtain spatial gene expression matrices and cell coordinates. Specifically, the GRCm39 genome was used as a reference. The DAPI-stained image was used for cell segmentation via the watershed algorithm implemented in CellPose v2.2.3.^107^ The cell segmentation data, which contained cells with distinct spots, were utilized to perform cell type annotation based on spatial transcriptomics.

#### Spatial transcriptome data analysis

To achieve spatial-level cell annotation, the annotation results from single-cell analysis were integrated, and the SpaceXR v2.2.1 package^108^ was utilized to predict the first and second cellular components of each spot. Gene set scores were calculated using the “AddModuleScore” function. The AddModuleScore scores for different cell types were visualized using the R package ggplot2 v3.5.1. The SpatialFeaturePlot function was used to generate spatial expression heatmaps.

### Human single-cell colon datasets

To investigate whether murine distal colonocytes have counterparts in human colonocytes, three human single-cell RNA sequencing datasets from E-MTAB-9543, E-MTAB-8007, and E-MTAB-8474, accessible at the Human Gut Atlas (https://gutcellatlas.org/pangi.html, accessed 15th January 2025), were used. When combined, these datasets contained gene expression data and metadata of 30 healthy colon samples, consisting of 36,390 healthy colon cells in total. Data normalization was performed on independent tissues using the variance-stabilizing transformation method implemented in the SCTransform function of Seurat. The datasets were subsequently integrated using the “IntegrateLayers” function with the method set to “CCAIntegration”. The method for determining the number of dimensions remained consistent with that described previously. The first 16 principal components (PCs) were employed to construct a shared nearest neighbor (SNN) network. A graph-based clustering approach, the Louvain algorithm, was then applied to identify cell clusters, with the resolution set to 0.3. Finally, UMAP was used to visualize the clustering results. To evaluate which cluster was more likely to be the distal colonocyte cluster (DCC), a featured gene set was obtained by employing the “FindAllMarkers” function. This gene set was subsequently filtered according to the criteria of an adjusted *p*-value < 0.05 and an average log_2_FC > 1. After filtering, the number of DCC feature genes was 235. These genes were programmatically converted to their corresponding one-to-one human orthologs by using the R package gprofiler2 v0.2.3^101^ and scored via the AddModuleScore function.

The second part of the dataset was the train.Epi.seur.rds file obtained from https://github.com/cssmillie/ulcerative_colitis.^109^ The dataset comprises two types of colon tissues: healthy colon tissue, designated “healthy tissue”, and ulcerative colitis (UC) colon tissue. The UC colon tissue can be further classified into “noninflamed tissue” and “inflammatory tissue”. The data encompassed a total of 49,765 cells, with 31,367 in the healthy group, 4,350 in the inflamed group, and 14,048 in the uninflamed group. The dataset was subsequently scored using the AddModuleScore function with the distal colonocyte cluster (DCC) gene set. Differential expression gene (DEG) analysis was subsequently conducted on this cell cluster. After that, the clusterProfiler package v4.6.2^104^ was utilized for gene ontology (GO) biological process term enrichment analysis. Significant terms were filtered based on an adjusted *p*-value < 0.05 and a log_2_FC > 1. Finally, the UpSetR package v1.4.0^110^ was used to quantify the intersection of significantly enriched GO terms between specific cells in the inflammatory and healthy tissues of UC patients and those of murine DCCs from S. Tm-infected mice. The heatmap package was subsequently used to plot the common terms.

### Statistical analysis and reproducibility

Statistical analysis is described in each figure legend. All data were collected from at least three independent experiments, unless otherwise indicated. The data were combined and are presented as mean ± SEM or median, with 25th and 75th percentiles as indicated. All image analyses were performed using R (R Core Team, Vienna, Austria, https://www.r-project.org/) v4.3.3 and Python v3.8.5 (Python Software Foundation, Delaware, USA, https://www.python.org/). The results were analyzed via various statistical tests using GraphPad Prism version 9.5.1 (GraphPad Software, Boston, Massachusetts, USA; www.graphpad.com), with *p* < 0.05 considered statistically significant. When presented in the figures, the significance levels are as follows: *p < 0.05; **p < 0.01; ***p < 0.001; and ****p < 0.0001; NS, not significant. The data distributions were assumed to be normal, but this assumption was not formally tested. The data were analyzed by Student’s t-test and one-way ANOVA. Tukey’s test was used for multiple comparisons. The correlation between two continuous variables was analysed by Pearson correlation. Survival data were analyzed via Kaplan‒Meier tests.

## Supplementary Material

Supplementary material Supplemental Figures

## Data Availability

The data supporting the findings of this study are available within the article and its supplementary files. Publicly available datasets can be accessed at the Gut Cell Atlas (https://www.gutcellatlas.org/). The published datasets are readily available through ArrayExpress with the accession numbers E-MTAB-9543,^47^ E-MTAB-8007,^111^ and E-MTAB-8474,^111^ which are also detailed in Table S11. The datasets generated during this study have been deposited in the Gene Expression Omnibus (GEO) database under accession codes GSE290763 (bulk RNAseq), GSE291248 (scRNAseq) and GSE290762 (spatial transcriptomics). All the codes used to generate the data in this study are available on GitHub (https://github.com/ZhangRuifen/Salmonella-typhimurium-targets-distal-colonocytes-to-trigger-host-responses). Where not otherwise included in this manuscript or its supplementary material, bacterial strains, original microscopy images and other relevant data are available from the corresponding author (Dan Xu, dan.xu@xjtu.edu.cn) upon reasonable request. Material transfer agreements may be required to distribute the resources and materials generated in this study.
