# Supplementary material for: Spatially resolved single-cell transcriptome analysis of murine Salmonella infection reveals the role of distal colonocytes in the inflammatory response
Source: Gut Microbes. 2025 Nov 24;17(1):2579909. doi: 10.1080/19490976.2025.2579909 (PMC12645889; doi:10.1080/19490976.2025.2579909)
Supplement: Supplementary material — Supplemental Figures [file KGMI_A_2579909_SM8199.docx]

**Supplemental Figures**

**
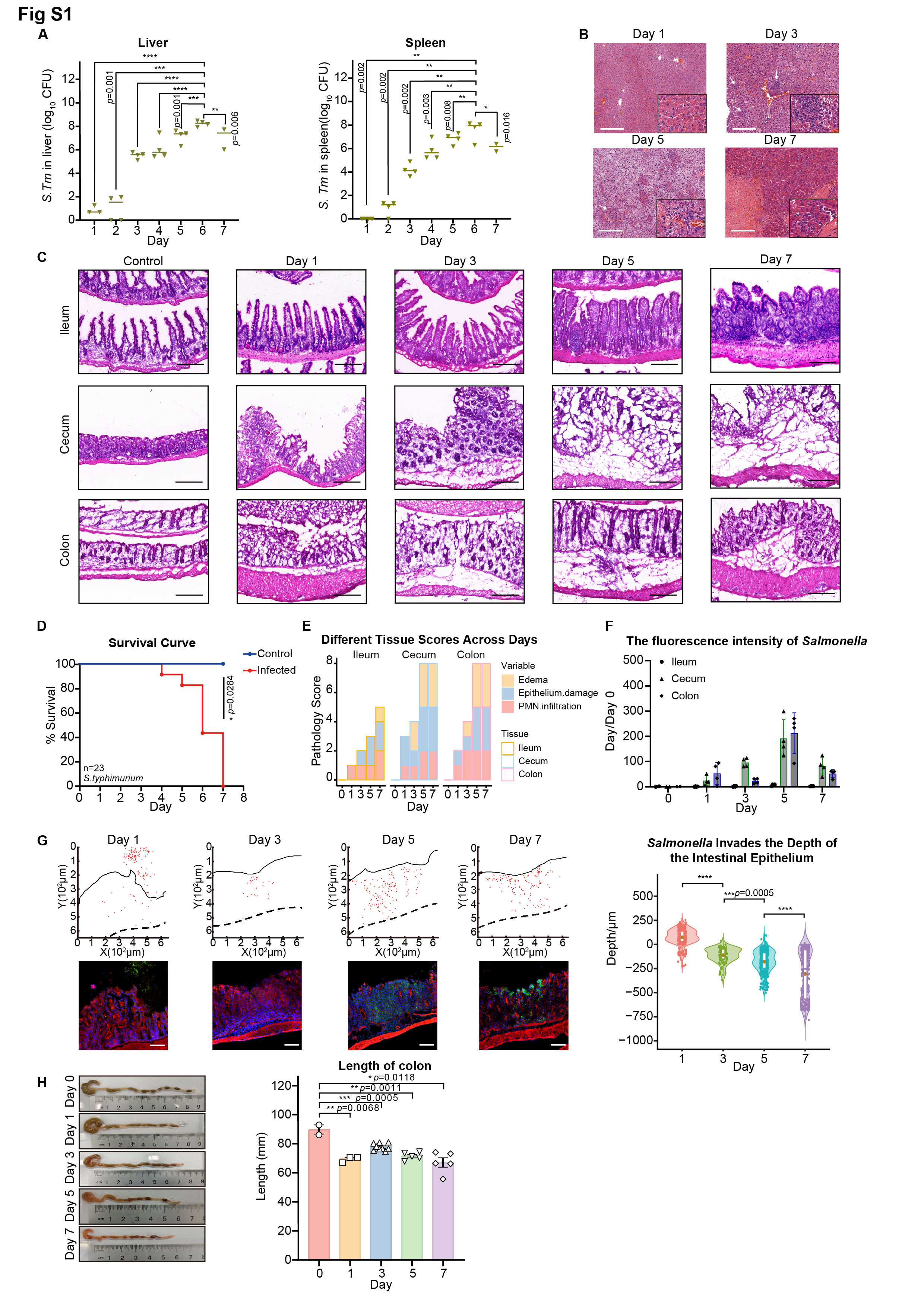
**

**Figure S1**. **S. Tm infection course in the murine model used in this study.** **(A)** The bacterial load in liver (left panel) and spleen (right panel) tissue of mice at different time points of S. Tm infection course (day 1, n = 3; days 2-6, n = 4; day 7, n = 2). **(B)** Histopathological analysis of liver collected from S. Tm*-*infected mice at different time points by hematoxylin and eosin staining. Inflammatory area is indicated by the white arrows. Scale bars, 200 μm. **(C)** Histopathological analysis of the ileum, cecum, and distal colon at different time points during the S. Tm infection course by hematoxylin and eosin staining. Scale bars, 100 μm. **(D)** Kaplan-Meier survival curve of mice with mock (n = 23) or S. Tm infection (n = 23). Significance was calculated by Kaplan-Meier test. **(E)** Quantification of histological inflammation scores in the ileum, cecum, and colon at different time points of S. Tm infection course. Data are shown as mean (n=5). **(F)** Relative fluorescence intensity of S.Tm in the ileum, cecum, and colon at different time points during the course of S.Tm infection. The Y-axis represents the ratio of fluorescence intensity at each time point to that of Day 0 (uninfected) for each segment. In order to quantify bacterial load in different intestinal segments after infection, intestinal tissue sections were subjected to immunofluorescent staining using FITC-conjugated anti-S. Tm LPS antibody. Fluorescence images were acquired with the same exposure settings. The fluorescence signal from each experimental group (different days post-infection) was normalized against uninfected controls. Data are presented as mean ± SEM. **(G)** Depth of invasion of S. Tm into distal colonic epithelium at different time points after infection in scatter plot (top left panel), immunofluorescence analysis (bottom left panel, scale bars, 100 μm, S. Tm shown in green, F-actin in red, nuclei in blue) and violin plots (right panel). Violin plots show the median, 25th and 75th percentiles, and whiskers extend to 1.5× the interquartile range. **(H)** The colon length of mice at different time points of S. Tm infection in appearance plot (left panel) and bar plot (right panel). Bar plots show mean ± SEM: each dot represents one mouse. (day 0, n = 2; day 1, n = 3; day 3, n = 9, day 5, n = 5; day 7 n = 5). Significance was determined by one-way ANOVA with Tukey’s post hoc adjustment, and asterisks represent statistical differences between indicated groups. **p* < 0.05, ***p* < 0.01, ****p* < 0.001, *****p* < 0.0001.


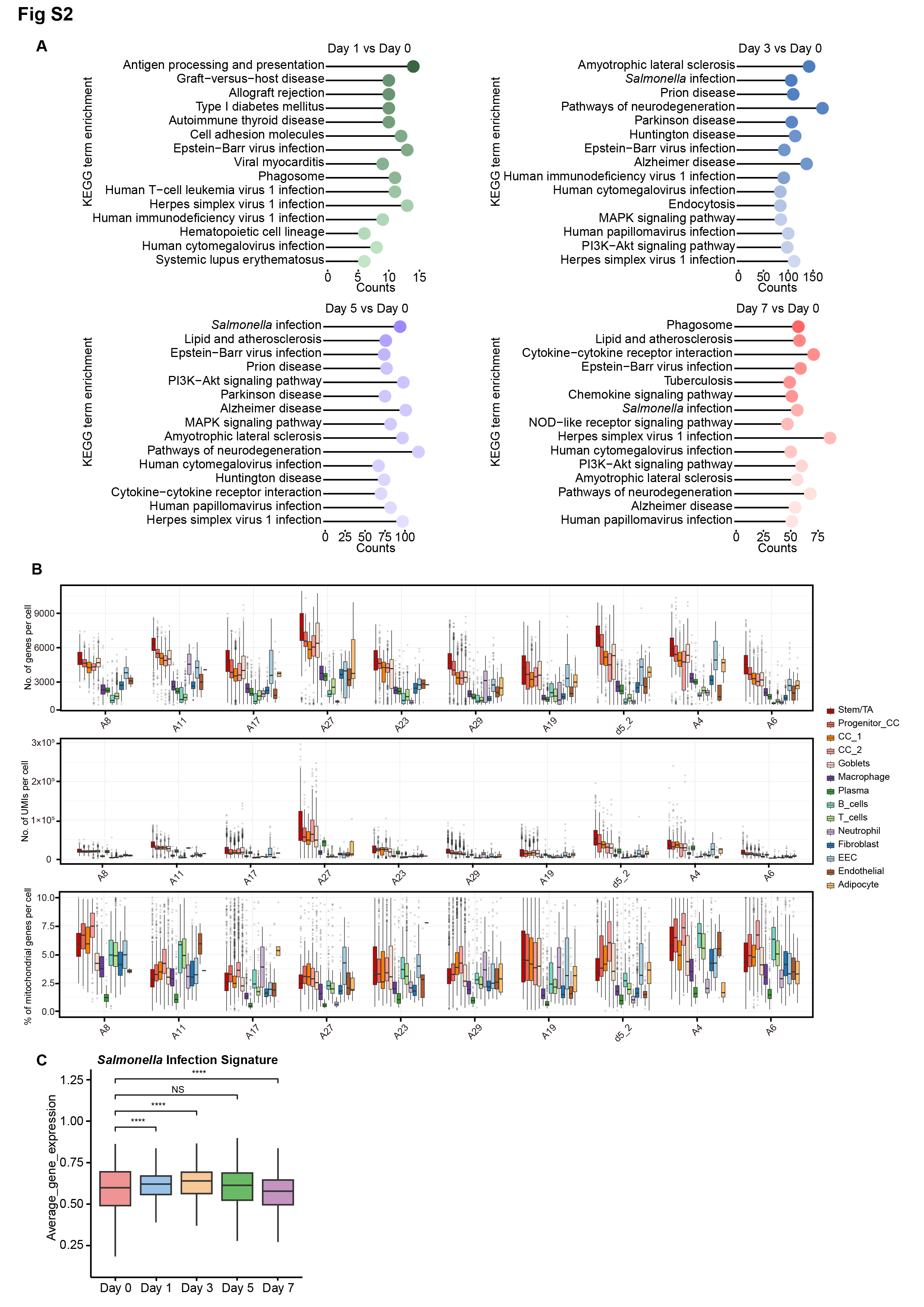


**Figure S2.** **KEGG enrichment analysis of** **bulk transcriptomic data and quality control of single-cell transcriptomic data. (A)** KEGG enrichment of DEGs obtained from bulk RNA-seq of colonic tissue at different time points after S. Tm infection relative to uninfected mice (the DEGs selected by adjusted *p*-value<0.05 and |log_2_FC|>1). **(B)** Box plots of the number of genes, number of UMIs and ratio of mitochondrial genes in each cell type of single-cell transcriptomes from different time points. **(C)** Box plot of average gene expression (after normalization) of the “*Salmonella* infection” gene set in single-cell transcriptome datasets at different time points. Box plots show the median, 25th and 75th percentiles, and whiskers extend to 1.5× the interquartile range. Significance was calculated by one-way ANOVA with Tukey’s post hoc adjustment, and asterisks represent statistical differences between indicated groups. NS, not significant; *****p* < 0.0001.

**
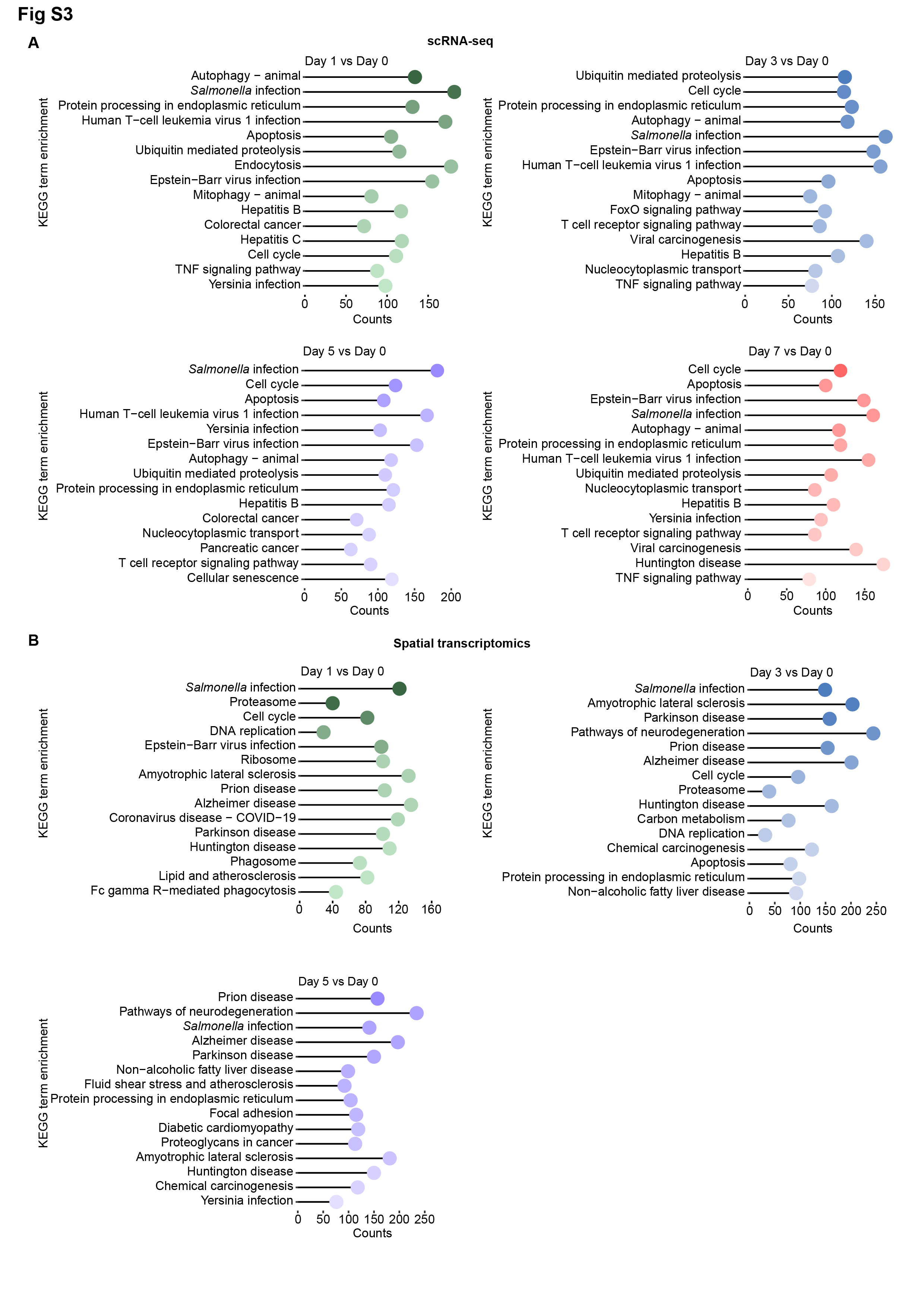
**

**Figure S3.** **KEGG enrichment analysis of single-cell and spatial transcriptomic data.** **(A)** KEGG enrichment of DEGs obtained in single-cell transcriptome of colonic tissue at different time points after S. Tm infection relative to uninfected mice (the DEGs selected by adjusted *p*-value<0.05 and |log_2_FC|>1). **(B)** KEGG enrichment of DEGs obtained in spatial transcriptome of colonic tissue at different time points after S. Tm infection relative to uninfected mice (the DEGs selected by adjusted *p*-value<0.05 and |log_2_FC|>1).


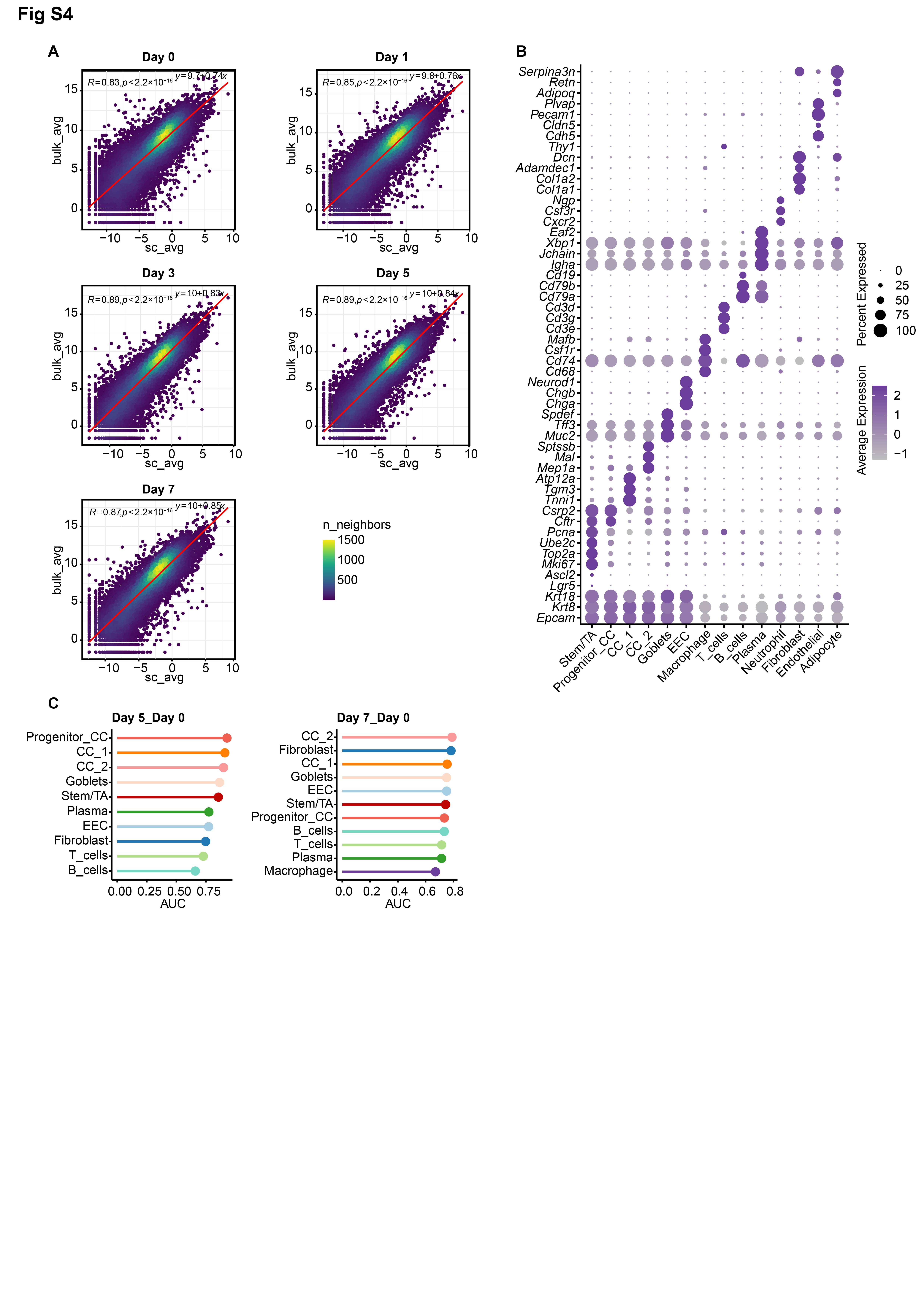


**Figure S4. Correlation and annotation of single-cell transcriptome and assessment of cell-type responsiveness to S. Tm infection.** **(A)** Correlation analysis of single-cell RNA-seq and bulk RNA-seq datasets at different time points of S. Tm infection. Significance was calculated by Pearson correlation. **(B)** Dot plots of the genes used to annotate cell populations. **(C)** Lollipop plot of the Augur AUC score for each cell type on days 5 and 7 post S. Tm infection.


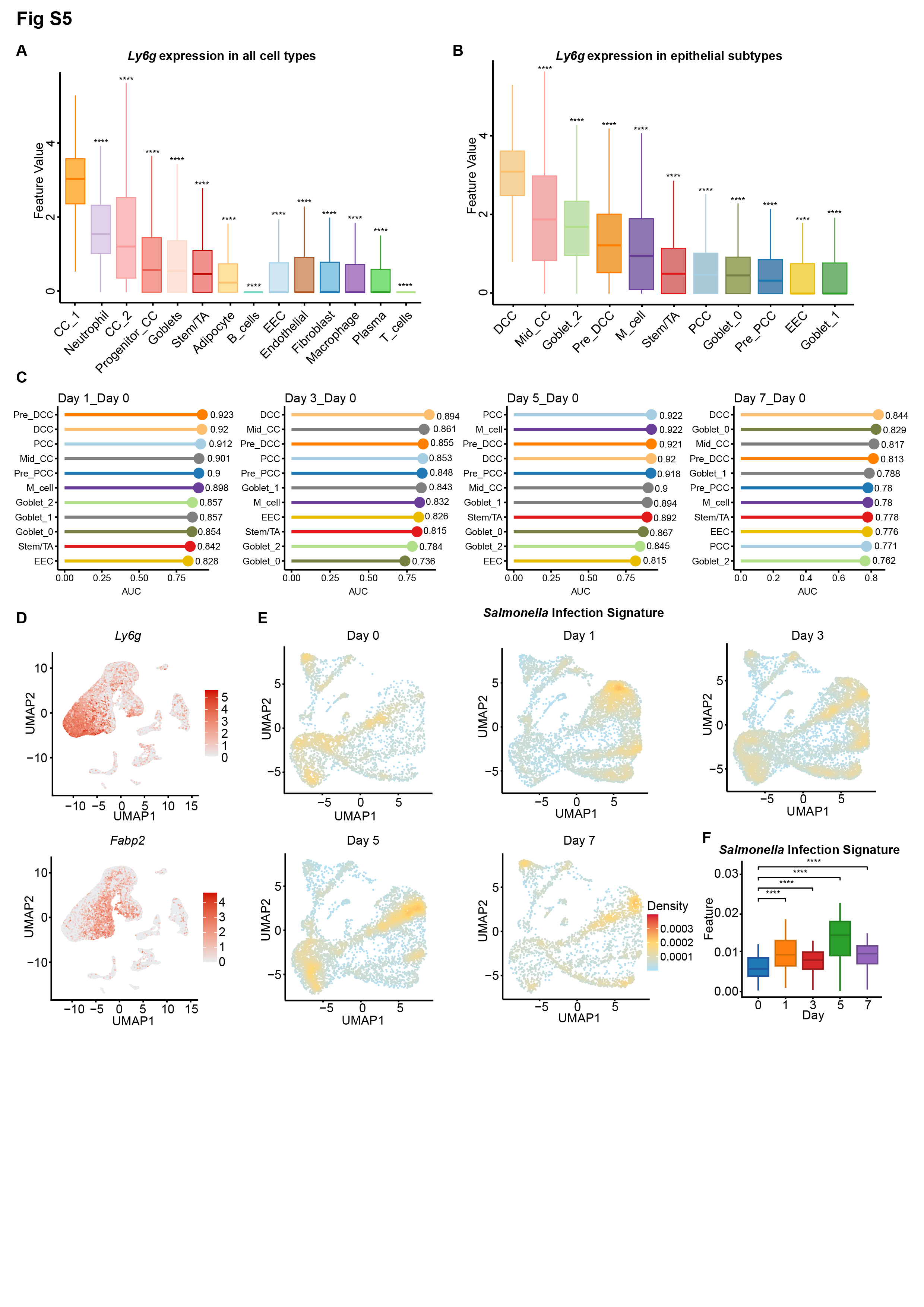


**Figure S5. Characterization of response of DCCs to S. Tm infection.** **(A, B)** Box plots showing *Ly6g* expression across all cell types **(A)** and epithelial cell subpopulations **(B)** using single-cell data pooled from all time points post S. Tm infection. **(C)** Lollipop plot of the Augur area under the curve (AUC) score for epithelial cell subpopulations to evaluate their response to S. Tm infection (AUC = 0.5 signifies no perturbation). DCC, distal colonocytes; PCC, proximal colonocytes; CC, colonocyte; TA, transit-amplifying cells; EEC, enteroendocrine cell. **(D)** Differential distribution of *Ly6g* (top panel) and *Fabp2* (bottom panel) expression in each cell type presented in UMAP plot. **(E)** irGSEA scoring of epithelial cell subpopulations in single-cell data using the gene set associated with S. Tm infection from bulk transcriptomic analysis. Enrichment was indicated by color. **(F)** Box plot of the scores of S. Tm infection signature using “*Salmonella* infection” gene set at different time points by irGSEA in DCCs. Box plots show the median, 25th and 75th percentiles, and whiskers extend to 1.5× the interquartile range. Significance was calculated by one-way ANOVA with Tukey’s post hoc adjustment, and asterisks represent statistical differences between indicated groups. *****p* < 0.0001.


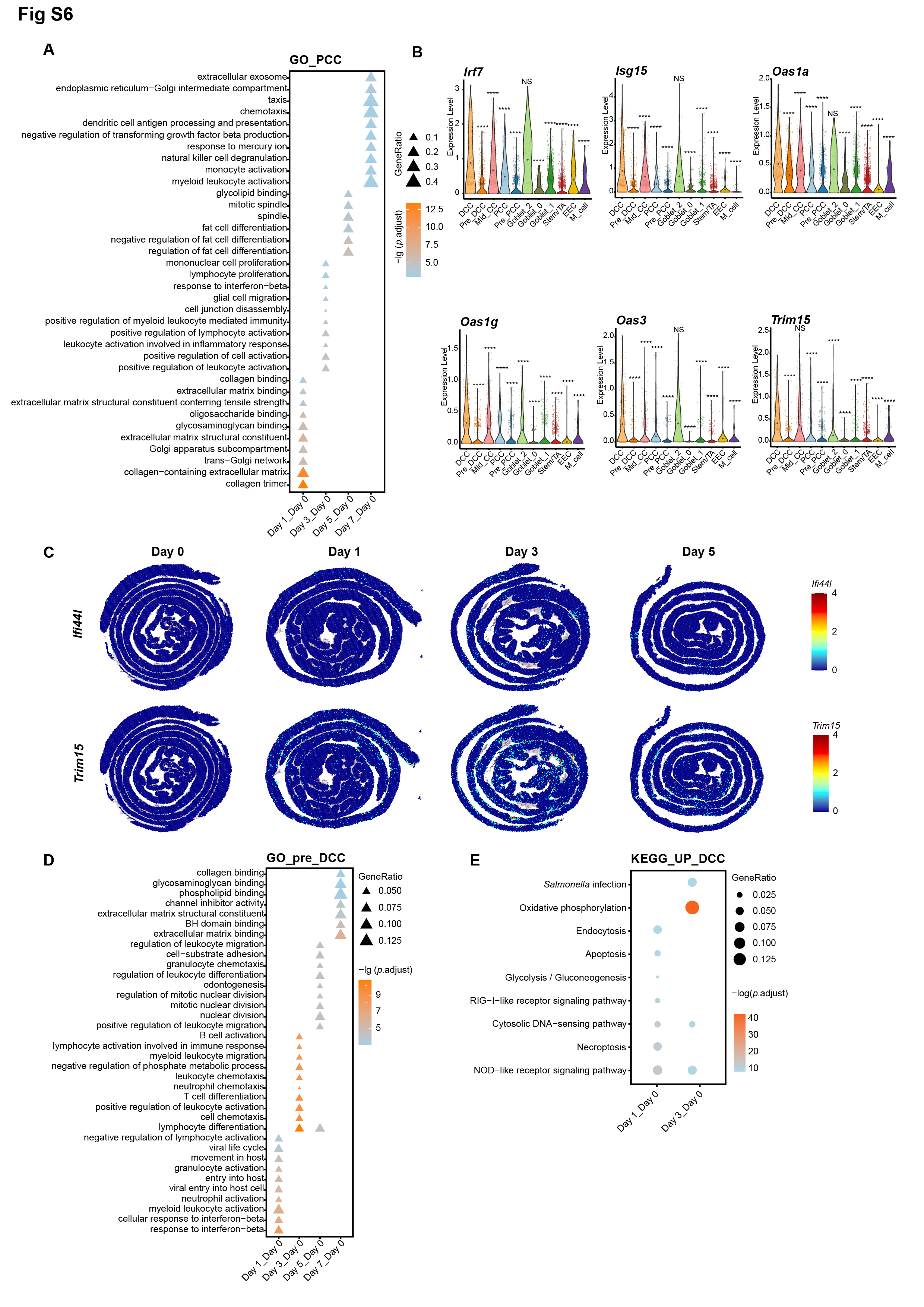


**Figure S6. Responsiveness of different epithelial cells to S. Tm and gene expression analysis of DCCs in naïve mice.** **(A)** GO enrichment analysis of DEGs in PCCs at different time points after S. Tm infection. The size of the dots represents the gene ratio of genes enriched to the term, and the colors represent -lg (*p*. adjust) value. **(B)** Violin plots showing expression of *Irf7, Isg15, Oas1a, Oas1g, Oas3* and *Trim15* in different epithelial cell subpopulations in naïve mice. Significance was calculated by one-way ANOVA with Tukey’s post hoc adjustment, and asterisks represent statistical differences compared with DCC. NS, not significant; *****p* < 0.0001. **(C)** Spatial distribution of *Ifi44l and Trim15* in the spatial transcriptome data at different time points. **(D)** GO enrichment analysis of DEGs in pre_DCCs at different time points after S. Tm infection. The size of the dots represents the gene ratio of genes enriched to the term, and the colors represent -lg (*p*. adjust) value. **(E)** KEGG enrichment analysis of upregulated pathways in DCCs at different time points post infection (day 1 and day 3). The size of the dots represents the gene ratio of genes enriched to each KEGG term, and the colors represent the -lg (*p*. adjust) value.


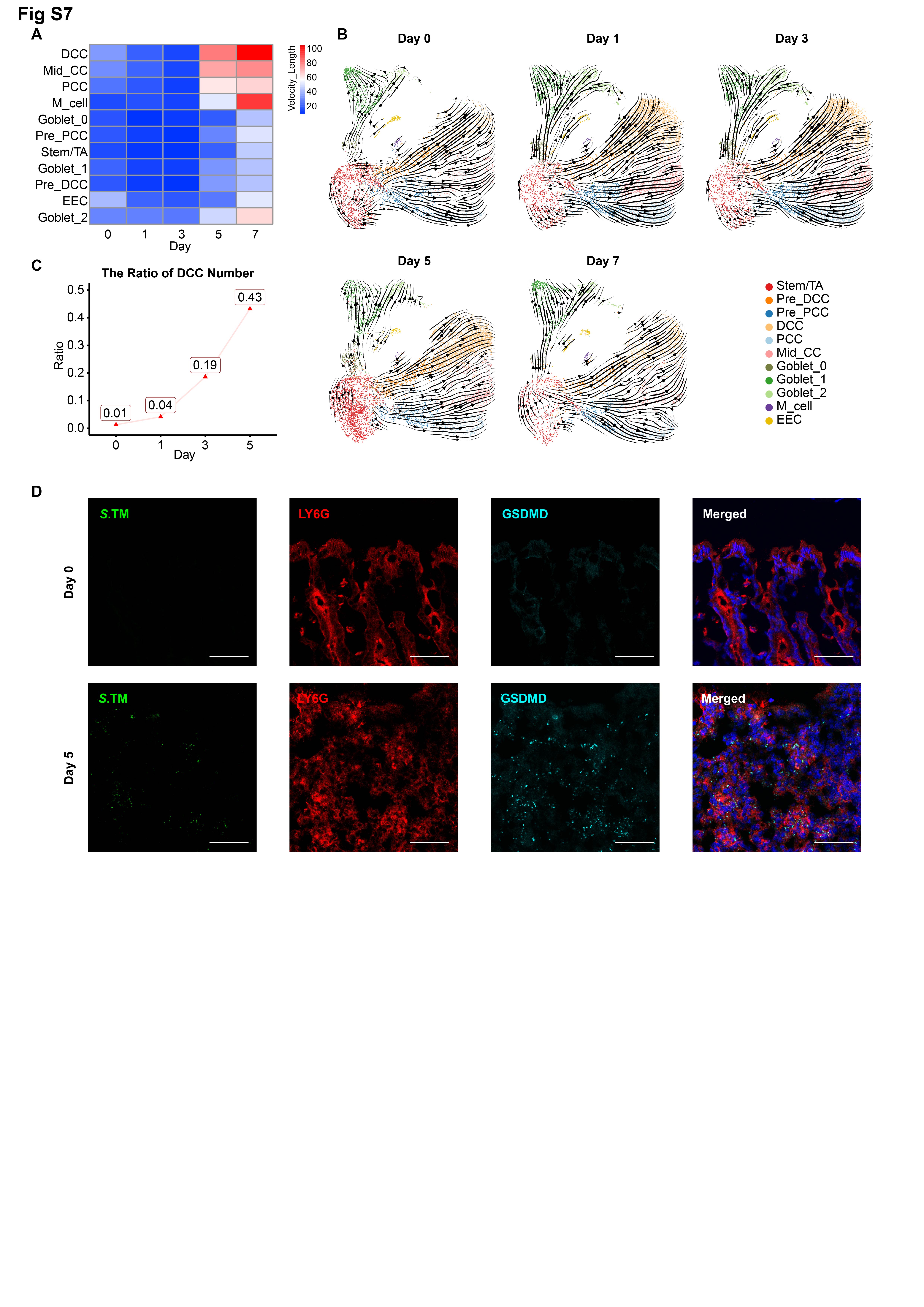


**Figure S7. Single-cell transcriptomic analysis and immunofluorescence analysis of DCCs during S. Tm infection.** **(A)** Velocity length of epithelial cell subpopulations at different time points of infection. Velocity length represents the length of the velocity vectors for each individual cell, with longer lengths indicating faster cell differentiation. **(B)** scRNA-seq velocity plots highlighting differentiation trajectory of colonic epithelial cells at different time points. DCC, distal colonocytes; PCC, proximal colonocytes; CC, colonocyte; TA, transit-amplifying cells; EEC, enteroendocrine cell. **(C)** The proportion of DCCs in spatial transcriptome data at different time points. **(D)** Immunofluorescence analysis of GSDMD expression in distal colonocytes before infection (day 0) and on day 5 post S. Tm infection. S. Tm bacteria are shown in green, LY6G in red, and GSDMD in cyan. Scale bars, 50 μm.


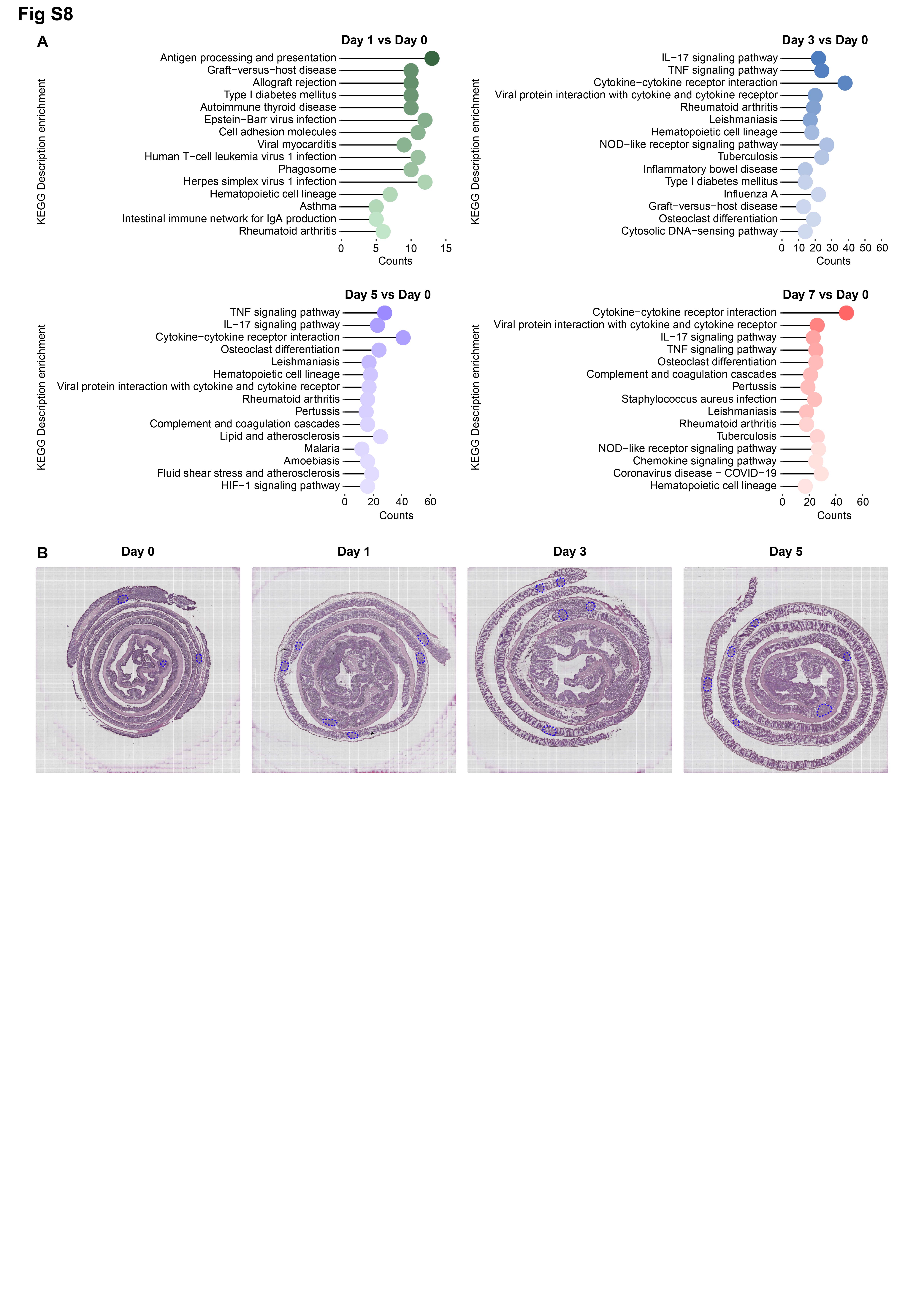


**Figure S8. Spatial transcriptome analysis of the colonic sections. (A)** KEGG enrichment of up-regulated DEGs obtained in bulk transcriptome of colonic tissue at different time points after S. Tm infection relative to uninfected mice (the DEGs selected by adjusted *p*-value<0.05 and log_2_FC>1). **(B)** Hematoxylin and eosin staining of colonic sections for the spatial transcriptome sequencing. The area circled by the blue dotted line is the mucosa-associated lymphoid tissue (MALT).


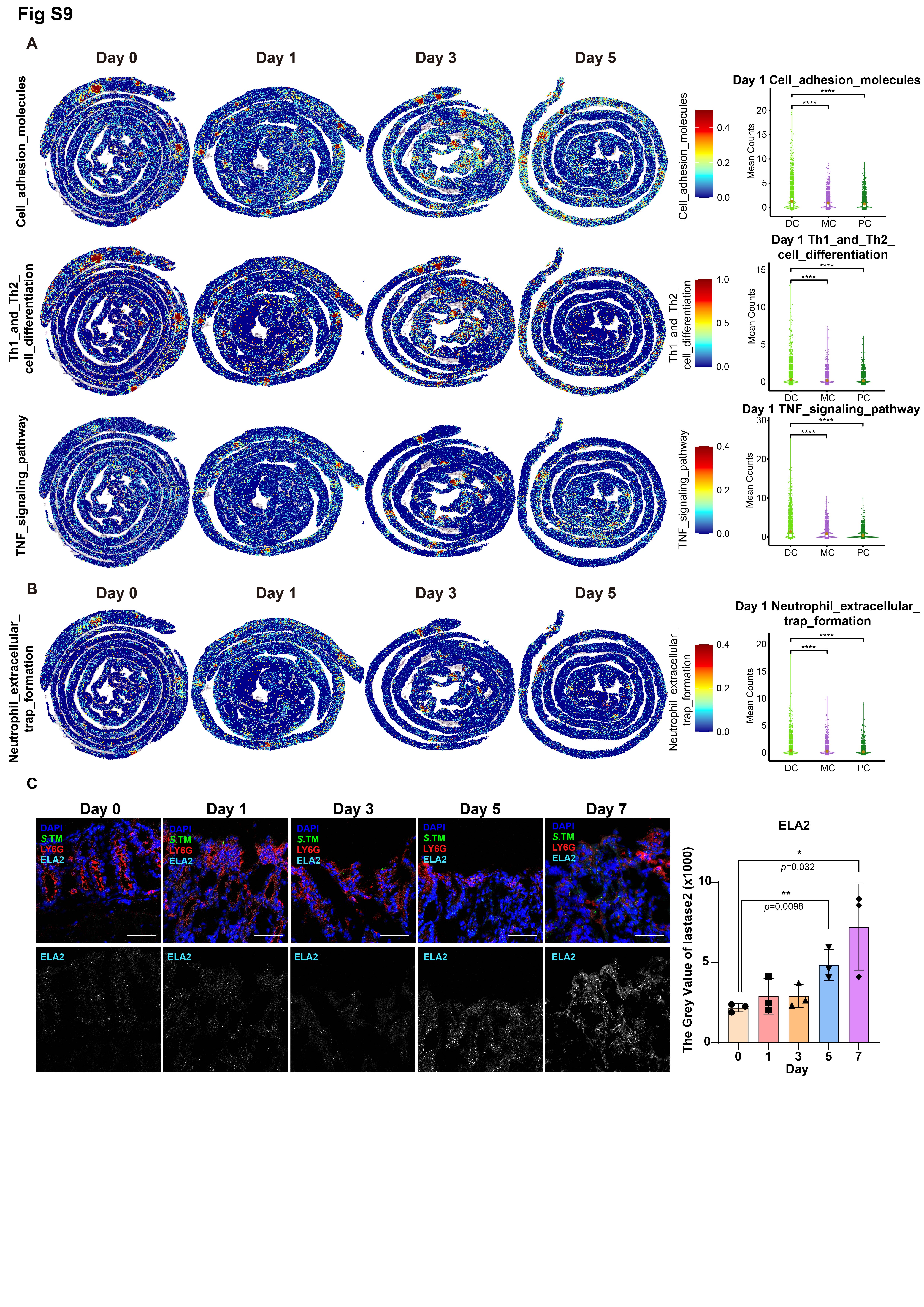


**Figure S9. Spatial distribution of activated inflammatory pathways and dynamic changes in ELA2 expression in the distal colon during S. Tm infection.**

**(A, B)** Spatial mapping of up-regulated “cell adhesion molecules”, “Th1 and Th2 cell differentiation”, “TNF signaling pathway” **(A)** and “neutrophil extracellular trap formation” **(B)** pathways upon S. Tm infection (left panels). Violin plots (right panels) showing the expression of the corresponding gene sets at day 1 in different colonic segments. Violin plots show the median, 25th and 75th percentiles, and whiskers extend to 1.5× the interquartile range. **(C)** Immunofluorescence analysis of ELA2 expression in distal colonocytes during the course of S. Tm infection (left panel), and corresponding quantification of ELA2 fluorescence intensity (right panel). S. Tm bacteria are green, LY6G is red, ELA2 is cyan, and nuclei are stained with DAPI (blue). Scale bars, 50 μm. Data are presented as mean ± SEM. Significance was calculated by one-way ANOVA with Tukey’s post hoc adjustment, and asterisks represent statistical differences compared with DC. **p* < 0.05, ***p* < 0.01, *****p* < 0.0001.


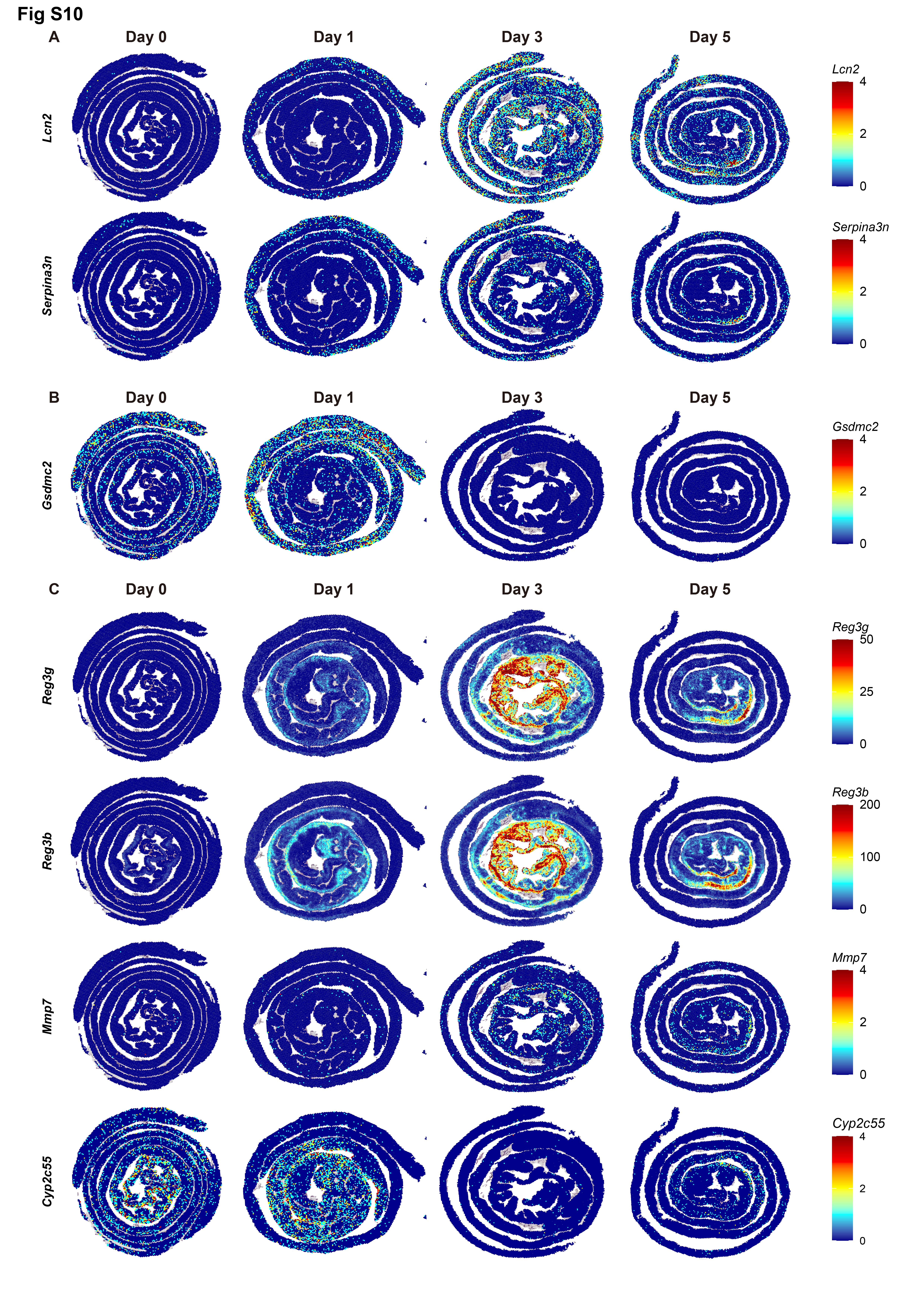


**Figure S10. Spatial mapping of genes that were significantly altered upon S. Tm infection.** **(A-C)** Spatial mapping of *Lcn2*, *Serpina3n* **(A)**, *Gsdmc2* **(B)**, *Reg3g*, *Reg3b*, *Mmp7* and *Cyp2c55* **(C)** expression in different colonic segments.


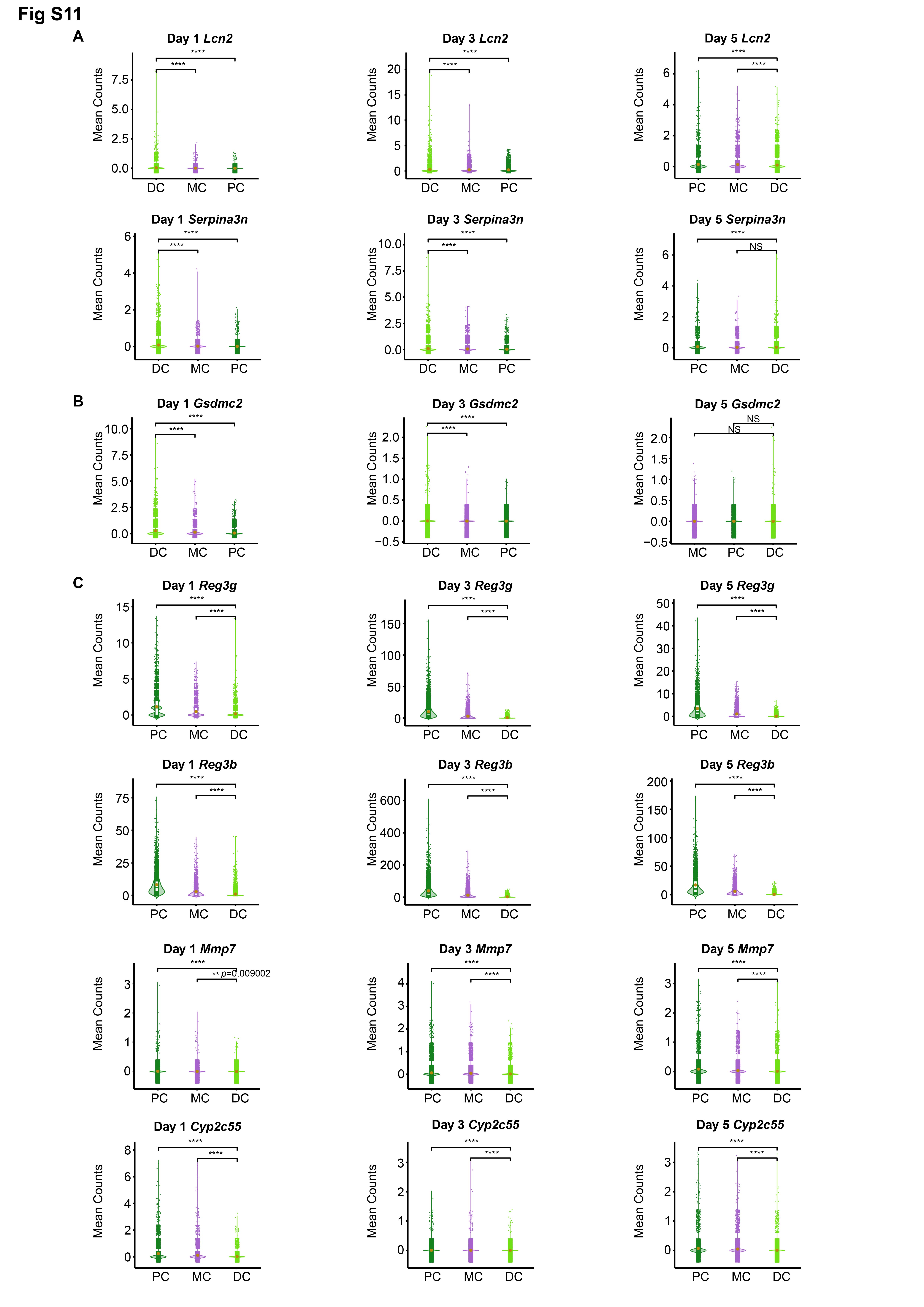


**Figure S11. Violin plots of distribution of genes that were significantly altered upon S. Tm infection in different colonic segments.** **(A-C)** Violin plots showing the expression of *Lcn2*, *Serpina3n* **(A)**, *Gsdmc2* **(B)**, *Reg3g*, *Reg3b*, *Mmp7* and *Cyp2c55* **(C)** expression at day 1, day 3 and day 5 in different colonic segments. Violin plots show the median, 25th and 75th percentiles, and whiskers extend to 1.5× the interquartile range. Significance was calculated by one-way ANOVA with Tukey’s post hoc adjustment, and asterisks represent statistical differences compared with DC (for **A-C**). NS, not significant; ***p* < 0.01, *****p* < 0.0001.


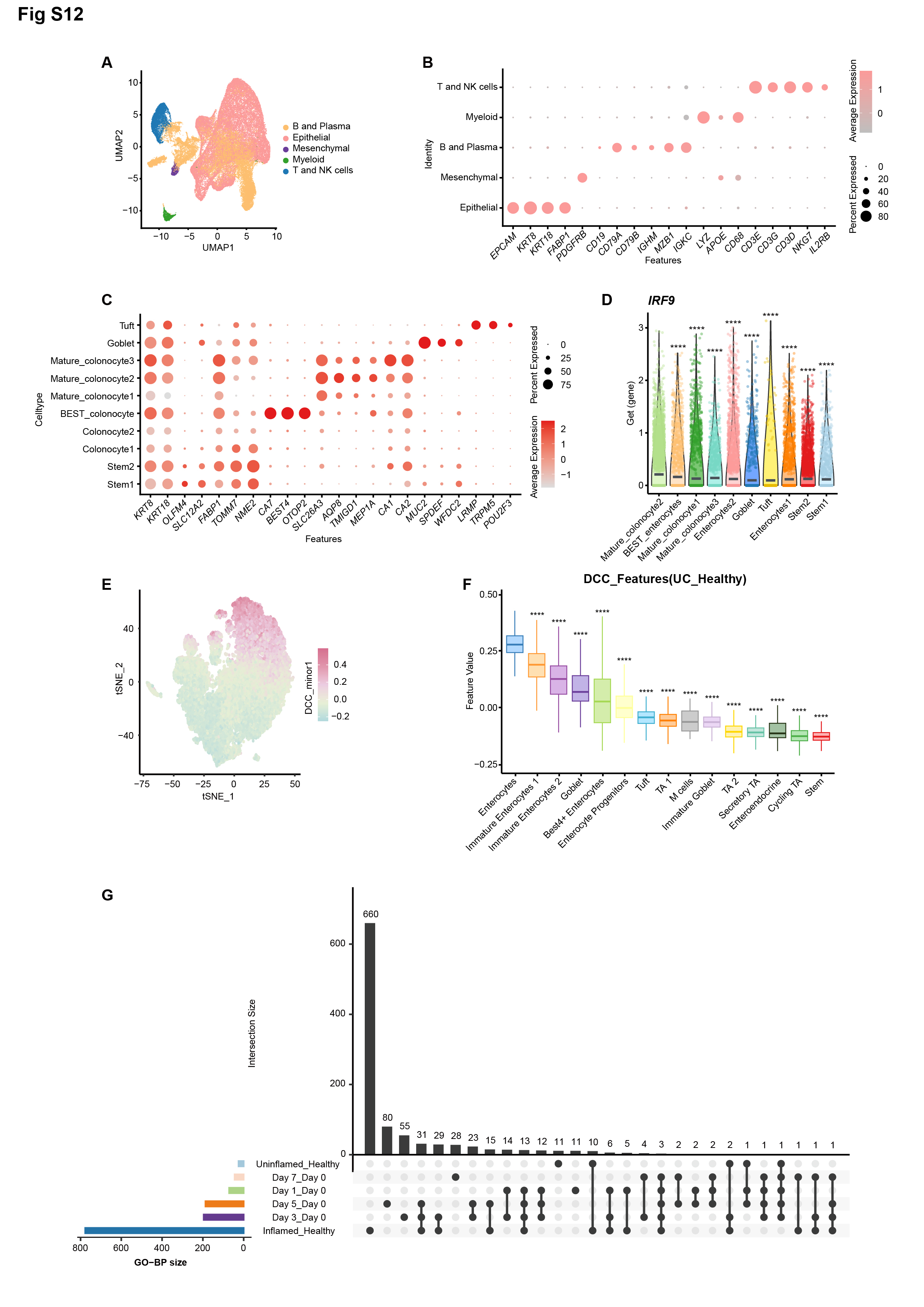


**Figure S12. Integrated analysis of human colon single-cell transcriptome datasets.**

**(A)** UMAP visualization of integrated single-cell transcriptomes of colon cells from healthy human colon samples extracted from the GCS (Gut Cell Survey) database. **(B)** Dot plots of the genes used to annotate human colon cell types. **(C)** Dot plots of the genes used to annotate colonic epithelial cell subpopulations. **(D)** Violin plots showing expression of *IRF9* in different human colonic epithelial cells. **(E, F)** *AddModuleScore* evaluation of human healthy colon epithelial cell subpopulations from UC data using murine DCC DEGs (genes differentially expressed in DCCs relative to all other colonic cell clusters, and selected by adjusted *p*-value<0.05 and log_2_FC>1). The color gradient in UMAP plot **(E)** represents the expression level of the gene set in each cell subpopulation. Detailed feature values are shown in panel **(F)**. **(G)** Upset plot showing intersections of GO terms in six sets. Top plot showing the size of intersections; left plot showing the size of six sets; main plot showing the origin of these intersections. Box plots show the median, 25th and 75th percentiles, and whiskers extend to 1.5× the interquartile range. Significance was calculated by one-way ANOVA with Tukey’s post hoc adjustment, and asterisks represent statistical differences compared with the mature colonocyte 2 (putative human DCCs) **(D)** or enterocytes **(F)** clusters. *****p* < 0.0001.
